# Insights into Parkinson’s Disease-Related Freezing of Gait Detection and Prediction Approaches: A Meta Analysis

**DOI:** 10.3390/s24123959

**Published:** 2024-06-18

**Authors:** Hagar Elbatanouny, Natasa Kleanthous, Hayssam Dahrouj, Sundus Alusi, Eqab Almajali, Soliman Mahmoud, Abir Hussain

**Affiliations:** 1 Department of Electrical Engineering, University of Sharjah, Sharjah 27272, United Arab Emirates; hdahrouj@sharjah.ac.ae (H.D.); ealmajali@sharjah.ac.ae (E.A.); solimanm@sharjah.ac.ae (S.M.); 2 O&P Electronics & Robotics Ltd., Limassol 3100, Cyprus; natasa.kleanthous@oprobotix.com; 3 The Walton Centre NHS Foundation Trust, Liverpool L9 7LJ, UK; sundus.alusi@nhs.net; 4 University of Khorfakkan, Khorfakkan, Sharjah 18119, United Arab Emirates

**Keywords:** Parkinson’s disease, FOG prediction, machine learning, explainable AI, wearable sensors, cueing devices

## Abstract

Parkinson’s Disease (PD) is a complex neurodegenerative disorder characterized by a spectrum of motor and non-motor symptoms, prominently featuring the freezing of gait (FOG), which significantly impairs patients’ quality of life. Despite extensive research, the precise mechanisms underlying FOG remain elusive, posing challenges for effective management and treatment. This paper presents a comprehensive meta-analysis of FOG prediction and detection methodologies, with a focus on the integration of wearable sensor technology and machine learning (ML) approaches. Through an exhaustive review of the literature, this study identifies key trends, datasets, preprocessing techniques, feature extraction methods, evaluation metrics, and comparative analyses between ML and non-ML approaches. The analysis also explores the utilization of cueing devices. The limited adoption of explainable AI (XAI) approaches in FOG prediction research represents a significant gap. Improving user acceptance and comprehension requires an understanding of the logic underlying algorithm predictions. Current FOG detection and prediction research has a number of limitations, which are identified in the discussion. These include issues with cueing devices, dataset constraints, ethical and privacy concerns, financial and accessibility restrictions, and the requirement for multidisciplinary collaboration. Future research avenues center on refining explainability, expanding and diversifying datasets, adhering to user requirements, and increasing detection and prediction accuracy. The findings contribute to advancing the understanding of FOG and offer valuable guidance for the development of more effective detection and prediction methodologies, ultimately benefiting individuals affected by PD.

## 1. Introduction

This section presents an overview of Parkinson’s disease, detailing the motivation and background that support this study. It further explains the problem statement and outlines the specific goals the work aims to achieve. Lastly, it describes the structure of the paper.

### 1.1. Motivation and Background

Parkinson’s disease (PD) stands as a significant neurological disorder characterized by an array of progressive symptoms identifiable through clinical diagnosis. It manifests through involuntary tremors, stiffness, bradykinesia, general muscle weakness, and rigidity. Among its most debilitating symptoms is the freezing of gait (FOG), a phenomenon causing sudden and unpredictable immobility in patients, contributing significantly to falls, reduced mobility, and overall decreased quality of life [1,2,3,4].

However, the scope of PD symptoms extends well beyond the motor domain. In 1817, James Parkinson first described the presence of non-motor symptoms in his seminal work “An Essay on the Shaking Palsy”, highlighting sleep disturbances, gastrointestinal issues, olfactory deficits, anxiety, and depression, which can precede motor symptoms by years, showcasing the complex and multifaceted nature of PD [4,5,6,7].

As PD progresses, motor functions and impairments deteriorate, influenced by factors such as age at onset, disease duration, and the involvement of non-dopaminergic brain regions [8]. The disease disrupts the supraspinal locomotor network—a neural system critical for locomotion control—due to diminished dopaminergic input to the striatum, affecting functionality across the locomotion circuit [9,10,11].

**Figure 1:**
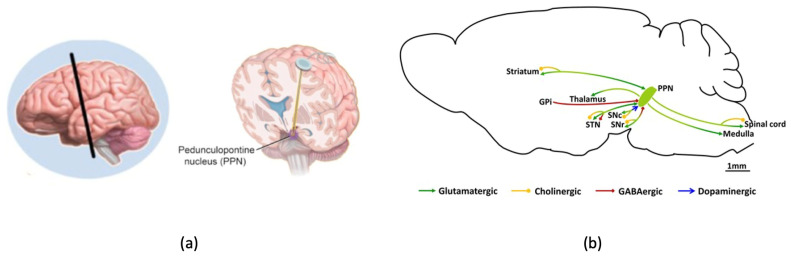
The structure of the PPN network. (**a**) The exact position of PPN [13]. (**b**) The connections of PPN neurons [12].

The midbrain motor area contains the pedunculopontine nucleus (PPN), shown in Figure 1a. It is a central hub that projects widely to a number of cerebral regions, including the thalamus, cortex, brainstem, cerebellum, and spinal cord as illustrated in Figure 1b [12,13]. Its critical function in regulating posture and gait is highlighted by its complex connection, especially with the basal ganglia [12,13]. According to Nutt et al. [14], FOG is directly linked to anomalies in PPN functional connectivity and microstructural irregularities within the subcortical area.

Wang et al. [15] further propose that the appearance of FOG in Parkinson’s disease is associated with disturbances in the functional connectivity networks of the PPN, particularly in the corticopontine–cerebellar pathways and visual temporal areas.

Globally, the prevalence of PD has doubled over the last 25 years, affecting over 8.5 million individuals in 2019, and is projected to increase further. This rise is mirrored in the Middle East and North Africa (MENA) region, with varying prevalence rates across countries [16,17]. Notably, PD primarily affects individuals over 50, though 4% of cases occur in younger populations. There is also a gender disparity, with men being 1.5 times more likely to be diagnosed with PD than women [18,19,20,21]. With the prevalence of PD increasing globally, the necessity for advanced management strategies is undeniable.

### 1.2. Problem Statement and the Purpose of the Work

The freezing of gait represents one of the most challenging and distressing symptoms faced by patients with Parkinson’s disease, profoundly impacting their quality of life [22]. Characterized by sudden, transient episodes of inability to move forward despite the intention to walk, FOG can lead to falls, fear of falling, and subsequent restrictions in daily activities, contributing to social isolation, and reduced life satisfaction [14,23]. The complexity of FOG, encompassing subtypes like start hesitation, turn hesitation, and destination hesitation, reflects its multifaceted impact on patients’ lives, exacerbating anxiety and reducing independence [24,25,26,27].

FOG manifests in various subtypes, each reflecting the symptom’s diverse impact on patients. These include (a) pure akinesia indicating an absence of leg movement, (b) trembling, characterized by an inability to move forward, accompanied by trembling at a rate of 2 to 4 Hz, and (c) shuffling, marked by a sudden increase in cadence alongside reduced step length [28,29,30].

The intricate nature of FOG, coupled with its unpredictable occurrences often triggered by multitasking, navigating tight spaces, or even changes in attentional focus, presents significant challenges for effective management and treatment [25]. Despite extensive research, the precise mechanisms underlying FOG remain only partially understood, complicating the development of targeted therapies and interventions [14,26,31,32]. In response to the limitations of conventional treatments, for the management of FOG in PD, cueing therapies have emerged as promising strategies [33]. These treatments employ external stimuli to facilitate walking, utilizing visual, auditory, or somatosensory cues to help patients initiate and maintain movement.

In recent years, machine learning (ML) and wearable sensor technologies have emerged as promising tools for the early detection and prediction of FOG episodes. By using the power of ML algorithms and the continuous monitoring capabilities of wearable devices, researchers have begun to develop systems capable of recognizing the precursors to FOG events, offering potential for real-time intervention and support. These advances hold the promise of transforming the management of FOG, enabling patients to engage in preemptive actions that may reduce the incidence or severity of freezing episodes, thereby enhancing mobility and autonomy [31,34,35,36].

Building upon this foundation, wearable cueing devices have emerged as a prominent application of these technologies, offering practical solutions for individuals with PD. These devices utilize external stimuli, such as visual, auditory, or somatosensory stimuli to aid in the initiation and maintenance of movement, directly addressing the challenges of FOG [33,37,38,39]. The aim of incorporating wearable cueing devices within this review is to underscore the practical applications of ML and sensor technologies, showcasing how they can be effectively employed to improve the quality of life for those affected by PD.

The goal of this work is to conduct a thorough review and analysis of the current landscape of FOG prediction and detection methodologies, with a particular focus on the integration of wearable sensor technology and ML approaches. Through a detailed examination and comparison of the existing studies, this review seeks to uncover gaps within the current body of research and highlight potential avenues for future investigations. This paper aims to guide the way to focused and patient-centered solutions for the management of FOG in PD, reflecting the ongoing commitment to improving patient outcomes through technological innovation.

The key contributions of this meta-analysis are multifaceted and pivotal to the advancement of freezing-of-gait research:

(a) It offers a comprehensive literature review, encompassing publications up to 2024. This review provides researchers and practitioners with a thorough understanding of the existing body of knowledge and serves as a valuable reference point for further investigation and study.
(b) It provides a description of the available cueing devices that can help unfreeze the gait of patients with PD.
(c) This analysis presents the available datasets relevant to FOG research, offering detailed descriptions of each dataset. By displaying the characteristics of these datasets, researchers can make informed decisions regarding data selection and utilization in their studies.
(d) The meta-analysis reveals the most common features extracted for FOG detection and prediction, showing the methodologies and techniques employed in feature extraction. This information equips researchers with the necessary insights to effectively extract and leverage features for FOG analysis.
(e) A critical aspect of this meta-analysis is the comparative assessment of ML versus non-ML approaches in terms of accuracy. By measuring p-values and z-values, this analysis quantitatively evaluates the performance disparity between these methodologies, providing intuitions into their relative efficacy and applicability in FOG research.
(f) Another facet is the exploration of the usage of explainable AI (XAI) within the context of FOG prediction. XAI offers a framework for creating transparent and interpretable ML models, facilitating greater understanding of FOG detection and prediction.

Together, these efforts enhance comprehension of FOG and lay the foundation for creating more efficient methods of detection and prediction, thereby benefiting individuals affected by Parkinson’s disease and related disorders.

### 1.3. Paper Organization

The paper is structured into several sections to provide a comprehensive analysis of the literature and findings related to freezing of gait detection and prediction. In Section 2, a review of relevant literature is presented, offering insights into the available datasets, existing knowledge, and cueing devices. Section 3 delves into detailed discussions covering various aspects such as datasets, preprocessing techniques, feature extraction methods, evaluation metrics, ML versus non-ML approaches, and the explainability of models, in addition to limitations and recommendations for future research directions. Finally, in Section 4, the conclusion section summarizes the study’s key findings and insights.

## 2. Study of the Literature

This section provides an overview of the selection criteria, an evaluation of the datasets used in the literature, an analysis of relevant literature, and a description of the available cueing devices.

### 2.1. Criteria for the Papers’ Inclusion and Exclusion

A search of IEEE Xplore, Elsevier, ACM Digital Library, PubMed, MDPI, Springer, and Google Scholar was performed in order to conduct a literature review. For FOG prediction and detection research, the terms “sensors”, “wearable device”, “Parkinson disease”, “freezing of gait”, “detect”, “predict”, and “pre-FOG” were utilized. After eliminating duplicates, the results were gathered for screening, with relevance assessed by utilizing keywords, abstracts, and publication titles. The papers were thoroughly examined after the screening. The use of sensor data as input, either directly from sensors or from readily available datasets, determined an analysis’ eligibility. The publications have to contain information from or involve PD patients who have experienced FOG. The analysis focused on 79 papers published between 2011 and 2024, excluding those published before 2011 or those that discussed solutions related to FOG but not detection or prediction. If an article was not in full text (only abstract) or was not published in English, it was excluded also.

The following characteristics were extracted from eligible publications, when available: year, population, type of the sensors, location of the sensors, features extracted, classifier or algorithm for FOG detection and prediction, window size, Pre-FOG duration, sampling rate, the aim of the paper, the performance and the source of the data utilized.

### 2.2. Datasets

The study of PD-related FOG benefits significantly from the availability of specialized datasets. Table 1 presents several key datasets such as Daphnet [40], IMU [41], and CuPiD [42,43]. These datasets are invaluable resources that consist of sensor data meticulously collected from individuals diagnosed with Parkinson’s disease. Each dataset provides a unique set of data points, enabling researchers to analyze and understand the intricacies of FOG in PD patients. Through these datasets, insights into the patterns, triggers, and variability of FOG episodes can be derived, offering a solid foundation for the development and testing of predictive models and technologies aimed at mitigating the impact of FOG on the lives of those affected by Parkinson’s disease.

**Table 1:** Description of the data available in the literature.

Dataset	# of Patients	Sensor Type	# of Sensors	Location of the Sensors	Created by
DAPHNet	10 patients	Accelerometer	3	Shank, thigh, and lower back	[40]
IMU	35 patients	IMU: gyroscope and accelerometer	-	Leg	[41]
Multimodal	12 patients	accelerometer, EEG, EMG, and skin conductance	-	Leg muscles, scalp, waist and arm	[44]
CuPiD IMU	18 patients	IMU: accelerometer and gyroscope	2	Above the ankle, and thigh	[43]
CuPiD multimodal	18 patients	IMU: accelerometer and gyroscope, ECG and SC	9, 1 and 1	Different parts of the body	[42]
BXHC	12 patients	Accelerometer, EEG, EMG, ECG, and SC	-	Different parts of the body	[45]
REMPARK	21 patients	IMU: accelerometer and gyroscope	1	Waist	[46]
6MWT	38 patients	Accelerometer and gyroscope	1	Lower back	[47]
ADL	59 patients	Accelerometer and gyroscope	1	Lower back	[48]

IMU: Inertial Measurement Unit, EEG: Electroencephalogram, EMG: Electromyogram, SC: skin conductance.

#### 2.2.1. Daphnet Dataset

Ten individuals with Parkinson’s disease participated in the Daphnet dataset [40], which was carried out at the Laboratory for Gait and Neurodynamics within the Department of Neurology at Tel Aviv Sourasky Medical Center (TASMC). Seven of the participants, whose average age was 66.5 years, were men. The accelerometer sensors on the wearable gadget that the researchers used were situated immediately above the ankle and knee, respectively, and were fastened with Velcro and elasticized straps. A third sensor was attached to the lower back. Eight hours and twenty minutes’ worth of data were made by all sensors using a 64 Hz sampling rate. Participants in the study completed two sessions: one with and one without real-time auditory cueing. The walking challenges included walking in straight lines, walking at random with stops and turns initiated by the user, and walking in diverse circumstances that mimic daily activities. In total, 237 FOG occurrences were recorded in video recordings that were synced with wearable device data. The average duration of these events ranged from 0.5 to 40.5 s.

#### 2.2.2. IMU Dataset

With permission from the local ethics council and consent from all participating Parkinson’s disease patients, the data collection for the IMU [41] dataset was carried out at the University of São Paulo, Brazil’s School of Physical Education and Sport. Thirteen male and sixteen female PD patients with FOG, ages ranging from 44 to 84 years, were selected from the University of São Paulo School of Medicine’s Movement Disorders Clinic. Measurements were made during the ON drug state to assure stability and consistency. The patients underwent three experimental sessions spaced one month apart to maximize the possibility of FOG occurrence. In order to collect data, experts in movement disorders conducted assessments using a variety of scales and conducted interviews to obtain clinical data, medication histories, and disease diagnoses. Wearing an inertial measurement unit (IMU) on the shank of the most affected body side, participants completed barefoot turning-in-place trials while recording triaxial linear accelerations and angular velocities at 128 Hz. The IMU consisted of a microelectromechanical device with sensors fastened to the shank with Velcro and elasticized straps. Video recordings of the turning trials were made, and specialist software was used to identify and analyze FOG incidents.

#### 2.2.3. Multimodal Dataset

The Ethics Committee at Xuanwu Hospital, Capital Medical University, Beijing, China, granted ethical approval for data collection, which was carried out at Beijing Xuanwu Hospital in accordance with the Declaration of Helsinki’s principles. In 2019, Beijing Xuanwu Hospital started collecting data [44], and 18 people in total who met the inclusion criteria and finished the process were included in the data. Among these, data from 12 subjects—six males and six females—were declared valid for an examination into the detection of FOG, including 13 sessions in which one subject conducted the experiment twice on different days. The age range of the individuals was 57–81 years, with an average age of 69.1 years. The length of the disease varied from 1 to 20 years, with an average of 9.3 years. During the trials, ten participants had noticeable FOG episodes. Using a multimodal sensory platform, electroencephalogram (EEG), electromyogram (EMG), gait acceleration (ACC), and skin conductance (SC) data were collected. A 32-channel wireless MOVE system was utilized to record EEG and EMG at a sampling rate of 1000 Hz. On the other hand, self-designed hardware subsystems based on TDK MPU6050 6-DoF accelerometers and gyros were used to acquire ACC and SC at a sampling rate of 500 Hz. A number of precise anatomical places were chosen for the sensors, including the left arm, the fifth lumbar spine (L5) of the waist, and the lateral tibia of the left and right legs.

#### 2.2.4. CuPid IMU Dataset

Important three-axis accelerometer and gyroscope data from inertial sensors mounted to patient wrists, recorded at 128 Hz, are available in the CuPiD IMU dataset [43]. This dataset contains measurements from eighteen patients, together with timestamps for Walking-with-Turns, Stops, and FOG events. With a mean age of 68.9 years and a standard deviation of 10.2 years, the subjects in the CuPiD IMU dataset range in age from 49 to 89 years. They have a PD diagnosis for a period of time ranging from 2 to 18 years, with a 4.6-year standard deviation and an 8.8-year mean.

In order to induce FOG events in a controlled setting, each patient completed walking activities including 180° and 360° turns in wide or narrow routes with obstacles during the data-collecting sessions. Furthermore, participants were trained to maneuver through congested hospital rooms. S1–S11, or 11 of the 18 patients, had a total of 184 FOG events labeled by the clinicians. The study excluded data from the seven remaining subjects who did not experience any episodes during the program. The information also includes intervals of time when patients stopped and conversed with physicians or chose to stop moving, denoted as “Stop”. The dataset contains labeled FOG events with durations ranging from 0.11 to 98.8 s, with a mean of 9.12 s and a standard deviation of 15.35 s. Interestingly, most incidents lasted less than 3 s (50.8%) and less than 5 s (64.7%). To properly detect these events, it is imperative to comprehend the range of FOG durations, which might impact the design of detection algorithms by specifying parameters such as window intervals and slide–step.

#### 2.2.5. CuPid Multimodal Dataset

The prediction power of ECG and SCR for FOG was examined using the CuPiD multimodal dataset, which comprises sensory data obtained from individuals with Parkinson’s disease. The ECG and SCR sensor data that make up the CuPid Multimodal dataset [42] were gathered in a laboratory setting during a variety of walking regimens that were intended to cause FOG. Actiwave1 was used to acquire the ECG data, collecting synchronized ECG and 3D acceleration data at a rate of 512 Hz. A Shimmer sensor2 was used to acquire the SCR data, sampling acceleration, and galvanic skin response (GSR) data at a rate of 51.2 Hz. Based on earlier studies, a motor activity protocol incorporating turns and navigating a small hallway was created to elicit FOG. Using two camera systems, FOG events were finely annotated offline, with physicians labeling the episodes based on observed gait patterns.

Eighteen PD participants, ages 49 to 89, with disease durations ranging from two to eighteen years, were included in the dataset. The lengths of the 184 FOG episodes, which ranged from 0.12 s to 98.88 s, were found. Subjects differed in the number of FOG occurrences they experienced; some had none, while others had over ten. A lesser percentage of FOG events had to do with starting a stride or walking straight ahead, while the majority happened during or after turning. Subject-to-subject heterogeneity was observed in FOG features and gait performance, which was probably impacted by medication status and contextual conditions.

#### 2.2.6. BXHC Dataset

FOG signals included acceleration data, EEG, EMG, ECG, and SC that were gathered from the BXHC dataset [45]. The study included a total of 12 people, whose ages ranged from 57 to 81 years, with an average age of 69.1 years, and whose disease duration varied from 1 to 20 years, with an average age of 9.3. A total of 88 min and 19 s were spent by 10 people in the trials experiencing 334 FOG episodes. Using a 100 Hz sample rate, two skilled clinicians classified the data from video recordings into two classes: 0 for the non-freezing state, and 1 for the freezing state.

#### 2.2.7. REMPARK Dataset

Data from 21 patients with PD who met certain inclusion criteria, such as having motor symptoms and a clinical diagnosis of PD, having a Hoehn and Yahr (H&Y) stage larger than two when therapy was not ongoing, and having a FOG questionnaire score (FOG-Q) greater than six, are included in the REMPARK dataset [46]. With an average age of 69.3 ± 9.7 years and an illness duration of 9 ± 4.8 years, the dataset consists of 18 males and 3 females. In the ON state, the MDS-UPDRS part-III total is 16.2 ± 9.7, while in the OFF state, it is 36.3 ± 14.4. The average H&Y score is 3.1 ± 0.4, the FOG-Q is 15.8 ± 4.1, and the Mini-Mental State Examination (MMSE) is 27.8 ± 1.9.

The studies were carried out in the homes of the patients, collecting data while the patients were receiving dopaminergic medication both ON and OFF. Various walking exercises and activities intended for false-positive analysis were among the tasks completed. An IMU with an elastic band around the left waist was used to record acceleration data. The sensor range was set at ±6 g, and the data were recorded at 200 Hz before being resampled to 40 Hz. During the period of the examinations, 9.1 h of inertial data were captured, including 93 min of FOG events.

#### 2.2.8. The 6 min Walking Test (6MWT) Dataset

Data from 21 control volunteers and 38 PD patients are included in the 6MWT dataset [47]. A clinical diagnosis of PD with motor symptoms, either with or without a history of FOG episodes, and no significant comorbidities or impairments impeding task performance were required for inclusion. PD subjects were in their regular ON condition, having taken their prescribed medicine, and the experiments were carried out at prearranged outpatient sessions. With an average age of 70.7 ± 8.2 years, an illness duration of 9 ± 4.8 years, and an H&Y score of 2.5 ± 0.8, the sample consisted of 10 females and 28 males. The control participants, consisting of 7 males and 14 females with an average age of 85.6 ± 7.2 years, were chosen based on the absence of symptoms of parkinsonism, severe visual impairment, dementia, or serious neurological problems. 6MWT involved the participants moving back and forth at their own pace along a 10-meter corridor. A smartphone-mounted 3-axis accelerometer and gyroscope, with a range of ±2 g and 2000 dps for the accelerometer and gyroscope, respectively, and a 200 Hz sampling rate, were used to gather the data. The smartphone contained local storage for inertial data. PD patients provided 2.4 h of inertial data during the tests, which included 17.4 min of standing, 5.3 min of FOG, and 97.6 min of gait. In addition, 1.4 h of data—72 min of gait and 4 min of stance—were collected from the control subjects.

#### 2.2.9. ADL Dataset

Data from 59 people with PD who satisfied the criterion for having PD-related motor symptoms without notable co-occurring conditions or disabilities that might have impacted their ability to complete tasks are included in the ADL dataset [48]. Everybody involved was in their regular everyday state. The average age of the group was 69.2 years, with an average disease duration of 6.7 years. There were 37 men and 22 women in the group. Their Hoehn and Yahr (H&Y) score, which gauges the severity of Parkinson’s disease, averaged 2.14. The sensor arrangement employed was similar to that of the 6MWT dataset. The trials took place during patients’ regularly scheduled visits to the doctor. During these visits, doctors gave participants instructions on how to move freely, turn at different angles, stand up, sit down, and complete tasks that were pertinent to the MDS-UPDRS evaluation. These semi-guided activities were supervised in a manner similar to what one might find in a typical home. In total, 5.9 h of inertial data were recorded during the course of the trials, which included 32.8 min of walking, 40.2 min of standing, and 13.5 min of postural alterations (e.g., sitting or standing up). The other tasks that were recorded included unmarked activities and elements connected to the MDS-UPDRS evaluation.

### 2.3. Related Work

Table 2 provides a summary of the literature that is currently available and offers information about the publication year, population, types and locations of the sensors, algorithm, features, window size, pre-FOG duration, sampling rate, aim, performance evaluation, and the source of the datasets.

### 2.4. Wearable Cueing Devices

Cueing devices provide external sensory signals or cues that can help “unfreeze” the gait of individuals with PD, facilitating smoother initiation and continuation of movement. These devices exploit the preserved ability of PD patients to respond to external rhythms or patterns, and are thought to contribute to FOG. Cueing can be delivered through various sensory modalities, including visual, auditory, and somatosensory (tactile) channels, each channeling different aspects of sensory processing to aid movement [30,39,49].

Visual cueing devices often project lines or patterns on the ground to guide step length and direction, leveraging the visual system’s role in spatial navigation and motor execution [37]. Auditory cueing devices, on the other hand, use rhythmic sounds or music to provide a temporal framework for stepping, engaging the auditory system’s capacity for rhythm perception and synchronization [50]. Somatosensory cueing devices apply tactile stimuli, such as vibrations or electrical pulses, to signal movement cues directly through the skin.

The development and implementation of cueing devices have been facilitated by advances in technology, allowing for the creation of wearable, portable, and user-friendly systems that can be customized to individual needs and preferences. These devices aim to reduce the frequency and severity of FOG episodes and enhance overall mobility, independence, and quality of life for people with PD.

While the exact neural mechanisms behind cueing are not fully understood, it is generally agreed that cueing shifts locomotor control from automatic processes to more goal-directed actions [51,52]. This shift involves increased activation of the prefrontal cortex (PFC) and other regions associated with attention and sensorimotor integration [53].

It is also reported that cueing can engage alternative neural pathways, bypassing the impaired basal ganglia. Visual cues, for instance, have been found to increase activity in the parietal and occipital cortices, as well as in corticocerebellar pathways, indicating their role in activating different motor control circuits. Similarly, auditory cues, such as rhythmic sounds, help synchronize steps by leveraging the brain’s inherent rhythm processing abilities. This synchronization can mitigate some of the timing dysfunctions associated with the basal ganglia [30,51,53].

**Table 2:** Detailed description of the available literature.

Reference	Year	Population	Type of Sensor	Location of the Sensor	Algorithm	Features	Window Size	Pre-FOG Duration	Sampling Rate	Aim	Performance	Source of the Dataset
[54]	2024	12 patients	IMU: Accelerometer + Gyroscope	3 sensors, on lateral tibia of both left and right legs, and fifth lumbar spine of waist (L5)	CNN	CNN features	0.5 s	1 s to 4 s	500 Hz	FOG prediction	NW: (Sens = 77.96%), (Spec = 89.90%), (prec = 78.33%) Pre-FOG: (Sens = 72.92%), (Spec = 85.44%), (prec = 72.52%) FOG: (Sens = 74.48%), (Spec = 87.30%), (prec = 75.13%) Accuracy: 75.02%	BXHC [45]
[55]	2024	(10 patients) (12 patients)	(Accelerometer) (EEG, EMG, ECG, SC, and acceleration)	(3 sensors, ankle, thigh, trunk) (left leg and wrist from acceleration sensor)	DL	Raw signal	0.5 s, 1 s, 2 s, 3 s	1 s, 2 s, 3 s, 4 s, 5 s	(64 Hz) (100 Hz)	3 class prediction, walking, FOG, and transition.	sensitivity of 84.61%, a specificity of 94.74%, and an F1 score of 86.19% for pre-FOG class	BXHC [45] + Daphnet [40]
[56]	2024	10 patients	Accelerometer	3 sensors, ankle, thigh, trunk	RF	Mean, median, mode, min, max, range, harmonic mean, standard deviation, variance, mean absolute deviation, median absolute deviation, kurtosis, skewness, root mean square, locomotory power, freezing power, freezing index, sum power, mean frequency, median frequency, spectral centroid, spectral kurtosis, spectral entropy, spectral peak	0.5 s	3 s	64 Hz	4 class prediction, No FOG, FOG, pre-FOG, post-FOG	Average accuracy of 96.5%	Daphnet [40]
[57]	2024	10 patients	Accelerometer	3 sensors, ankle, thigh, trunk	DL	Freezing Index, information entropy, Teager energy entropy, the frequency with a ratio of cumulative energy in the band, the sum of the power in the freezing band (3–8 Hz) and the locomotion band (0.5–3 Hz), mean, and standard deviation	1 s	1 s, 3 s, 5 s	64 Hz	3 class prediction, walking, FOG and Pre-FOG.	Accuracy of 95.40% with an MF1 score of 0.89 and a Kappa coefficient of 0.87.	Daphnet [40]
[58]	2024	12 patients	IMU: Accelerometer +Gyroscope	The pelvis and both sides of the tibia and talus	LSTM, TCN, SVM, KNN, XGBoost	65 features	1 s, 2 s and 4 s	-	64 Hz	FOG Detection	FOG Intra-class correlation coefficient of 95%	-
[59]	2024	1170 patients	Accelerometer	Lower back	Bagging and stacking, LightGBM as the base estimator	Mean, median, minimum, maximum, andstandard deviation.	5 s	-	128 Hz	FOG Detection	MAP score of 0.306	Michael J. Fox Foundation for Parkinson’s Research
[60]	2023	(10 patients) and (35 patients)	(Accelerometer) and (IMU)	(3 sensors, ankle, thigh, trunk) and (leg)	Different combinations of parallel CNN Networks.	CNN Features	2 s to 4 s	-	64 Hz	FOG Detection	98.1% with Daphnet dataset and 98.6% with IMU dataset	Daphnet [40] + IMU [41]
[61]	2023	20 Patients	IMU: Accelerometer +Gyroscope	7 sensors: waist (1), thigh (2), calf (2), ankle (2)	Autoregressive (AR) model and SVM	Mean, variance, standard deviation, max, min, energy, interquartile range, range, entropy, DC component, skewness, kurtosis of amplitude and shape.	0.5 s	-	100 Hz	FOG prediction	accuracy of 85.08%	-
[62]	2023	-	Accelerometer	-	BiLSTM	The mean, maximum, minimum, and standard deviation	-	-	124 Hz	FOG prediction	combined score of 0.427	DeFOG Competition [63]
[64]	2023	8 patients	EEG and IMU	Head, left forearm, left shank	NN	Raw signal	1 s, 2 s, 3 s, 4 s, 5 s, 6 s	-	500 Hz	FOG prediction	accuracy of 92.1%	
[65]	2023	a. 21 patients b. 38 patients c. 59 patients	a. IMU: Accelerometer + Gyroscope b. Accelerometer +Gyroscope c. Accelerometer +Gyroscope	a. 1 sensor: waist b. 1 sensor: lower back c. 1 sensor: lower back	CNN	CNN features	2 s	-	a. 200 Hz downs-ampled to 40 Hz b. 200 Hz c. 200 Hz	FOG Detection	specificity above 88%	a.REMPAR-K dataset [46] b. 6MWT dataset [47] c. ADL dataset [48]
[44]	2022	12 patients	Accelerometer, EEG, EMG, and SC	lateral tibia of the left and right legs, fifth lumbar spine (L5) of the waist, left arm, muscle of the right leg, tibia anterior (TA) muscles of both legs, second belly of the left index finger and middle finger.	SVM model	Wavelet energy, wavelet entropy, mean absolute value, zeros crossing, slope sign change, wave length, three direction accelerations, the associated sample entropy, standard deviation, total power, freezing index, median value, minimum value, and maximum value.	3 s	-	EEG and EMG at 1000 Hz (downsampled to 500 Hz), accelerometer and Sc at 500 Hz	FOG prediction	All combinations exceeded 93% accuracy.	Multimodal dataset [44]
[66]	2022	12 patients	Accelerometer Gyroscope Force sensing resistor sensors	7 sensors: Waist (1), Thigh (2), Shank (2), Sole (2)	Threshold	Freeze Index, energy, sum power, mean, absolute mean, zero crossing rate, standard deviation, range, root mean square, maximum, minimum, principal direction eigenvalue, entropy	2 s	-	100 Hz	FOG Detection	sensitivity 78.39%, specificity 91.66%, accuracy 88.09%, precision 77.58%, f-score 77.98%	-
[67]	2022	11 patients	Plantar pressure sensor	2 sensors: at soles	Decision tree and random undersampling boosting	Number, duration, length of COP reversals, number, duration, length of COP deviations, CV of COP position, velocity, acceleration, number of weight shifts, power, Dominant Frequency, max, min, mean, Freeze Index, variance, and energy	1 s	2 s	100 Hz	FOG prediction (Binary, (Pre-FOG) and the freeze episodes were in the target class)	sensitivity 77.3%, specificity 82.9%	[68]
[69]	2022	12 patients	IMU: Accelerometer +Gyroscope EEG	3 sensors: Waist on L5 (1) Leg (2)	LSTM	Freezing index, sample entropy, energy index; standard deviation.	2 s	-	1000 Hz downsampled to 500 Hz	FOG Detection	geometric mean 91.0%	-
[70]	2022	63 patients	IMU: Accelerometer +Gyroscope	3 sensors: Ankle (2), 7th cervical vertebra (1)	CWT and CNN	cadence, step duration, velocity, stride length, FOG Criterion, gait cycle duration (stride time, stance time and swing time), power in the freezing band (between 3 and 8 Hz) and locomotor band (0.5 to 3 Hz).	2.56 s	-	50 Hz	FOG Detection	geometric mean 90.7%, F1 score 91.5%, sensitivity 91.9%, specificity 89.5%	-
[71]	2022	16 patients	IMU: Accelerometer +Gyroscope	6 sensors: Chest (1), Lumbar region (1), Ankle (2), Feet (2)	CNN	CNN features	2 s	-	64 Hz	FOG Detection	AUC 83%	-
[72]	2022	7 patients	IMU: Accelerometer +Gyroscope	2 sensors: ankle	CNN, Transfer learning, and k-mean clusters	CNN features	2 s	0.5 s, 1 s	128 Hz	FOG Prediction	sensitivity 63.0%, specificity 98.6%	[73]
[74]	2022	12 patients	EEG, EMG, ECG, EOG, SC, IMU: Accelerometer +Gyroscope	4 sensors: Lateral tibia of the leg (2), Fifth lumbar spine (1), Wrist (1)	SVM and KNN	Total Power, Mean Power, Max Power, STD Power, Locomotion Band Power, Freeze Band Power, Locomotion Band Power STD, Freeze Band Power STD, Freeze Index, Freeze Ratio, Skewness, Kurtosis, Energy, Entropy, Dominant Frequency, Mean Frequency, Median Frequency, RMS, Mean, STD, Number of zero-crossing, Zero-crossing rate, Number of peaks, Mean distance between peaks, Mean height of the peaks, Energy, Max Amplitude, Min Amplitude, Range, Integral, Axes correlation, Total Power, Mean Power, STD Power, Max Power, Dominant Frequency, Mean Frequency, Median Frequency	3 s	-	500 Hz	FOG prediction	subject-independent accuracy 85%	-
[34]	2021	11 patients	inertial sensor (IMU)	2 sensors: on each shin,	Binary classification: DT, SVM PreFOG: SVM, LDA, KNN, LR	Standard Deviation, Range, Root Mean Square, Angular Jerk, Normalized Jerk, Stride Similarity, step time, stride time, Peak height, Peak width, Power Spectral Entropy, Principal Harmonic Frequency, Principal Harmonic Amplitude, Principal Harmonic Width, Weighted Power Spectral Frequency, Low Power Frequency	2 s to 5 s	2 s to 5 s	60 Hz	FOG and Pre-FOG detection.	FOG and PreFOG respectively Se 95.9%, 84% Sp 95.4%, 88.3% Ac 95.5%, 87.4%	-
[75]	2021	10 patients	Pressure sensors Accelerometer Angular velocity Sensor Euler angles sensor	2 sensors at soles	CNN+ANN	CNN features	0.5 s	-	50 Hz	FOG Prediction	sensitivity 96.0%, specificity 99.6%, precision 89.5%, accuracy 99.5%	-
[76]	2021	10 patients	accelerometer	3 sensors: ankle, thigh, trunk	KNN, SVM and MLP	time-domain attributes + PCA	0.94 s	-	64 Hz	no FOG, pre-FOG, FOG, and pre of post FOG prediction	precision of no FOG, pre-FOG, FOG, and pre of post FOG is 99.42%, 92.23%, 97.84%, and 92.73%, respectively, accuracy of 98.92%	Daphnet [40]
[77]	2021	28 patients	3D motion analysis	Anatomical landmarks were marked with 34 retroreflective markers.	CNN + LRP to provide transparency.	CNN features	-	-	100 Hz	explainable FOG prediction	Accuracy of 86%	[78]
[79]	2021	11 patients	Plantar pressure sensors	insoles	LSTM networks	Center of pressure (COP), COP velocity, COP acceleration, Ground Reaction Force (GRF) and Fraction of total GRF	1 s	2 s	100 Hz	FOG prediction Binary, (Pre-FOG) and the freeze episodes were in the target class)	2-layer LSTM: 82.1% sensitivity and 89.5% specificity. 3-layer LSTM: 83.4% sensitivity and 87.4% specificity.	[68]
[80]	2021	10 patients	Accelerometer	3 sensors: ankle, thigh, trunk	LSTM network	Raw signal	1 s	-	64 Hz	FOG Detection	AUC score of 97.62%	Daphnet [40]
[81]	2021	18 patients	IMU: Accelerometer +Gyroscope	wrist	CNN	CNN features	0.25 s	-	128 Hz	FOG Detection	90% specificity and 86% sensitivity	CuPiD IMU Dataset
[82]	2021	10 patients	Accelerometer	3 sensors: ankle, thigh, trunk	LSTM	Raw signal	0.5 s, 1 s, 2 s, 3 s	1 s and 3 s	64 Hz	FOG prediction: They used preFOG to predict FOG	89% prediction accuracy	Daphnet [40]
[83]	2021	10 patients	Accelerometer	3 sensors, ankle, thigh, trunk	CNN and LSTM	Min, max, range, mean, median, mode, trimmed mean, standard deviation, variance, root mean square, mean absolute value, median absolute deviation, 25th percentile, 75th percentile, interquantile range, normalized signal magnitude area, skewness, kurtosis, mean crossing rate, signal vector magnitude, peak of Fourier transform, entropy, energy, peak frequency, Freeze Index, band power, RP, STFT, DWT and PWVD	1 s to 4 s	1 window	64 Hz	FOG Detection and Prediction	accuracy 98.5%, sensitivity 98.5%, specificity 97.9%	Daphnet [40]
[68]	2021	11 patients	Plantar pressure sensor	2 sensors: at soles	decision tree ensemble	Number, duration, length of COP reversals, number, duration, length of COP deviations, CV of COP position, velocity, acceleration, number of weight shifts, power, Dominant Frequency, max, min, mean, Freeze Index, variance, and energy	1 s	2 s	100 Hz	FOG, pre-FOG and transition between FOG and pre-FOG detection	76.4% sensitivity and 86.2% specificity	[68]
[84]	2021	10 patients	Accelerometer	3 sensors: ankle, thigh, trunk	LDA, regression trees, SVM and RF.	Norm, max, min, median, incidence coefficient and degree of proximity.	-	-	64 Hz	FOG Detection	accuracy 89.94%, sensitivity 87.8%, specificity 93.02%	Daphnet [40]
[85]	2020	10 patients	accelerometer	3 sensors: ankle, thigh, trunk	CNN + The attention-enhanced LSTM	CNN features	-	-	64 Hz	FOG Detection	95.1% sensitivity and 98.8% specificity	Daphnet [40]
[86]	2020	12 patients	Accelerometer	Lower back	AdaBoost	Freezing Index, variance, Dominant Frequency, cadence, step regularity, and gait pattern variability	5 s	5 windows	100 Hz	FOG prediction	Model A: 77%, model B: 81.2%, model C: 80.9%, model D: 82.7%	-
[87]	2020	10 patients	accelerometer	3 sensors: ankle, thigh, trunk	LDA and PCA	variance, mode, standard deviation, maximum and minimum values	1 s to 6 s	2 s to 4 s	64 Hz	FOG prediction: They used preFOG to predict FOG	FOG: sensitivity and specificity of 94.1% and 97.1%, respectively.	Daphnet [40]
[88]	2020	1 patient	IMU: Accelerometer +Gyroscope	2 sensors: on both shanks	Closed-loop DBS algorithms	arrhythmicity over the last six steps (AR), stride time (ST), swing angular range (SA), and asymmetry over the last six steps (AS)	-	-	128 Hz	FOG Prediction	-	-
[89]	2020	67 patients	IMU: Accelerometer + Gyroscope	3 sensors: Ankle (2), 7th cervical vertebra (1)	CNN	CNN features	4 s	-	50 frames per second	FOG Detection	accuracy 89.2%, geometric mean 88.8%	-
[90]	2020	10 patients	Accelerometer	3 sensors, ankle, thigh, trunk	RF, GB, XGB, SVM, and NN	Mean, standard deviation, min, max, Quartile1, Quartile3, median, skew, kurtosis, zero crossing rate, peak-to-peak, crest factor, root mean square (RMS), velocity root mean square, entropy, Freeze Index, power difference, fast Fourier transform mean magnitude, fast Fourier transform mean phase, power spectrum, integrals, center of gravity of x, y, z components, angles of x, y, z components	2 s, 3 s, 4 s	2 s, 3 s, 4 s	64 Hz	3 class prediction: walking, FOG, and transition	FOG: sensitivity 72.34% specificity 87.36% Transition: sensitivity 91.49% specificity 88.51% Normal activity: sensitivity 75% specificity 93.62%	Daphnet [40]
[87]	2020	10 patients	Accelerometer	3 sensors: ankle, thigh, trunk	KNN	KLDA	1 s to 6 s	1 window	64 Hz	FOG prediction (2 classes: FOG and No FOG), (3 classes: FOG, Pre-FOG and No FOG)	specificity 97.1%, sensitivity 94.1%	Daphnet [40]
[91]	2020	71 patients	Accelerometer Gyroscope Magnetometer	4 sensors: Lower back (2), Ankle (2)	SVM	Mean standard deviation, correlations, range, RMS, peak amplitude, entropy, freezing index, power, skewness, kurtosis	3 s	-	128 Hz	FOG Detection	accuracy 85.0%, specificity 83.4%, sensitivity 84.1%	-
[92]	2020	21 patients	Accelerometer	1 sensor: waist	RNN, CNN and LSTM	128 features, 192 features and 256 features.	3.2 s	-	200 Hz downsampled to 40 Hz	FOG Detection	mean AUC 93.9%, mean specificity 87.1%, mean sensitivity 87.1%	-
[93]	2019	10 patients	accelerometer	3 sensors, ankle, thigh, trunk	Random orest, multilayer percep-tron and hidden Markov models, CNN+MLP	mean, standard deviation, variance, frequency entropy, energy, Freeze Index (power of the freeze band (3–8 Hz) divided by power in locomotor band (0.5–3 Hz)), and power in both bands, median absolute deviation, largest value, smallest value, signal magnitude area, interquartile range, ecdf, auto regression coefficients, the correlation coefficient between two axes, weighted average, skewness, kurtosis, Harmonicity in time and frequency domains, predictability in time and frequency domains, and spectral flux	4 s	-	64 Hz	FOG Detection	sensitivity 95%, specificity 75%	Daphnet [40]
[94]	2019	10 patients	accelerometer	3 sensors: ankle, thigh, trunk	Statistical	Freeze Index, the wavelet index and sample entropy	2 s, 2.5 s, 3 s, 3.5 s and 4 s.	2 s	64 Hz	FOG Prediction: They used preFOG to predict FOG	88.8%, 92.5%, and 89.0% for average predictivity, sensitivity, and specificity, respectively	Daphnet [40]
[95]	2019	7 Patients	EMG and IMU: accelerometer and gyroscope	Right leg	FOG detection algorithm	Absolute value of the averaged angular velocity, threshold, step window	-	-	6.6K Hz	FOG Detection	2% false negative and 5% false posi-tive	-
[96]	2019	10 patients	Accelerometer	3 sensors: ankle, thigh, trunk	autoregressive moving average model	Freeze Index, high-resolution time–frequency spectral	6 s	-	64 Hz	FOG Detection	sensitivity 99.2%, specificity 94.59%	Daphnet [40]
[97]	2019	18 patient	Accelerometer	2 sensors: ankle	Adaptive Synthetic sampling algorithm	Freeze Index, entropy, power, standard deviation	2 s	2 windows	128 Hz down sampled to 64 Hz	FOG Prediction	accuracy 97.4%, prediction 66.7%	[24]
[98]	2019	10 patients	Accelerometer	3 sensors: ankle, thigh, trunk	SVM and probabilistic neural networks	freezing index, extended freezing index, peak, average peak distance, number of dominant peaks, average peak width, zero crossings, zero crossings of jerk, variance, norms of acceleration, dominant singular values,	4 s	-	64 Hz	FOG Prediction	sensitivity 93%, specificity 91%	Daphnet [40]
[73]	2019	10 patients	Accelerometer Gyroscope Magnetometer	1 sensor: wrist	Threshold	Freeze Index	3 s	-	512 Hz	FOG Detection	accuracy 99.7%	-
[99]	2019	25 patients	IMU: Accelerometer +Gyroscope	2 sensors: ankles	NN	Freeze Index, freeze band, locomotor band, mean frequency, Dominant Frequency, power, spectral entropy, root mean square, mean, standard deviation, coefficient of variation, kurtosis, maximum acceleration, range of acceleration, stride peak, stride time, velocity, stride length, and the FOG criterion.	-	-	50 Hz	FOG Detection	specificity 93.1%, sensitivity 95.9%	-
[100]	2018	8 patients	accelerometer	3 sensors: ankle, thigh, trunk	MLP, RF, XGB, SVM, KNN, NB	mean, RMS velocity, proportion above mean, proportion below mean, sum of changes, madogram, variogram, peak frequency, Freeze Index	5 s	5 s	64 Hz	3 class prediction: walking, FOG, and transition.	W, F, T respectively Se: 86.00%, 89.00%, 75.00% Sp: 95.00%, 91.00%, 88.00% Ac: 91.00%, 90.00%, 82.00%	Daphnet [40]
[101]	2018	16 patients	EEG	head	Combination of DTF, ICA and Bayesian neural network	the mean, the maximum and the minimum values of DTF	5 s	5 s	500 Hz	FOG Prediction	Sensitivity of 82.65% and a specificity of 86.60%	-
[102]	2018	51 patients	Accelerometer	2 sensors: on both knees	SVM, k-NN, NB, DT	step time, stride time, step length, stride length, walking speed, standard deviation, harmonic ratio, and cross-correlation coefficient	-	-	32 Hz	FOG Detection	sensitivity and specificity of 90.89% and 91.2%, respectively	-
[103]	2018	21 patients	accelerometer, gyroscope and magnetometer	1 sensor: waist	DL	the mean, standard deviation, variance, the entropy, and the energy	2.56 s	-	50 Hz	FOG Detection	accuracy 89%, sensitivity 91.9%, specificity 89.5%	-
[104]	2018	15 patients	Accelerometer	1 sensor: waist	Threshold	means, difference among mean values, standard deviations, correlations, frequency standard deviation, highest harmonics and center of mass, skewness, kurtosis, integrals, auto-regression coefficients, principal component values	0.8 s, 1.6 s, 3.2 s, 6.4 s	-	40 Hz	FOG Detection	sensitivity 91.7%, specificity 87.4%	-
[105]	2017	10 patients	Accelerometer Gyroscope Magnetometer	3 sensors: Head (1), Ankle (2)	Threshold	Raw signal	1 s	-	50 Hz	FOG Detection	Accuracy: 92.86%	-
[106]	2017	10 patients	accelerometer	3 sensors: ankle, thigh, trunk	Time frequency domain analysis	Energy, freezing index and spectral coherence.	0.1 s to 1 s	-	64 Hz	FOG Detection	The accuracies were 92%, 90.1%, and 89.8% for the knee, ankle, and hip sensors.	Daphnet [40]
[107]	2017	11 patients	IMU Electrocardiography Skin-conductance	3 sensors: left and right ankle (2), and lower back (1)	Linear Discriminant Analysis	Turning degrees, gait symmetry (left–right cross-correlation, left–right difference in SD), gait amplitude, power in the locomotor band, power in the freezing band, and freezing index.	2 s	2 s	128 Hz	Pre-FOG Prediction	Good performance	Cubid dataset [42]
[108]	2017	10 patients	Accelerometer	3 sensors, ankle, thigh, trunk	Anomaly score detector with adaptive thresholding	maximum and number of peaks in the spectral coherence, Freezing index, Average, standard deviation, variance, median, entropy, energy, power	2 s to 8 s	-	64 Hz	FOG Detection	Ankle: (accuracy: 94%) (specificity: 84%) Lower back: (accuracy: 89%) (specificity: 94%)	Daphnet [40]
[83]	2017	16 patients without FOG and 28 patients with FOG.	IMU: Accelerometer +Gyroscope	2 sensors: on each shin	Ad hoc algorithms	Step velocity, stride length, stride time, and cadence.	-	-	25 Hz	FOG Detection	accuracy 98.51%, sensitivity 93.41%, specificity 98.51%	-
[46]	2017	21 patients	IMU: Accelerometer + Gyroscope (only accelerometer used)	1 sensor: Waist	SVM	Mean, Increments of consecutive windows’ mean values, Difference between the increments of the windows’ mean values, Standard deviation, Correlation, Highest harmonic peaks, Spectral density center of mass, Skewness, kurtosis, A change of basis, Integrals, Auto regression coefficients	3.2 s	-	200 Hz down sampled to 40 Hz	FOG Detection	sensitivity 74.7%, specificity 79.0%	-
[109]	2017	6 patients	EEG	1 sensor: head	Bayesian Neural Networks and time–frequency Stockwell Transform	maximum amplitude for each band at a time (t), the sum of amplitude of the each band at a time (t)	1 s	-	512 Hz	Turning FOG Detection	sensitivity 84.2%, specificity 88%, accuracy: 86.2%	-
[110]	2017	12 patients	IMU: Accelerometer + Gyroscope magnetometer	1 sensor: waist	SVM	mean, standard deviation, range, signal magnitude area, signal correlations, skewness, kurtosis; energy and spectral density in specific bands	-	-	40 Hz	FOG and Bradykinetic Gait Detection	sensitivity 82.08%, specificity 93.75%	-
[111]	2017	32 patients	Acceleromete Gyroscope	2 sensors: shin	Threshold	k-index	-	-	25 Hz	FOG Detection	accuracy 97.56%, precision 89.55%, sensitivity 93.41%, specificity 97.57%	-
[112]	2016	3 patients	accelerometer	3 sensors: shank, thigh, and torso	LRN	Raw signal	-	-	Approx. 200 Hz	FOG prediction	89% precision 30% recall	-
[111]	2017	32 patients	Acceleromete Gyroscope	2 sensors: shin	Threshold	k-index	-	-	25 Hz	FOG Detection	accuracy 97.56%, precision 89.55%, sensitivity 93.41%, specificity 97.57%	-
[112]	2016	3 patients	accelerometer	3 sensors: shank, thigh, and torso	LRN	Raw signal	-	-	Approx. 200 Hz	FOG prediction	89% precision 30% recall	-
[113]	2016	18 patients	IMU: Accelerometer +Gyroscope	2 sensors at ankles and 2 sensors at wrist	Supervised machine learning	Mean, Standard deviation, power	3 s	-	128 Hz	FOG Detection	Subject-dependent accuracy 85%, specificity 80%; subject-independent accuracy 90%, specificity 66%	[43]
[114]	2016	10 patients	Accelerometer	3 sensors, ankle, thigh, trunk	continuous wavelet transform (CWT)	Freeze Index	1 s, 2 s, 3 s, 4 s	-	64 Hz	FOG Detection	sensitivity: 82.1% specificity: 77.1%	Daphnet [40]
[115]	2016	20 patients	Accelerometer	1 sensor: Hip	Threshold	Freezing index, energy, and step cadence	2.56 s	-	100 Hz	FOG Detection	sensitivity 87.57%, specificity 94.97%	-
[42]	2015	18 patients	IMU, ECG and SC sensors	2 sensors: chest (1), finger (1)	Threshold	mean, median, variance, power on very low frequencies, power on low frequencies, power on high frequencies, ratio between the power, standard deviation, minimum, maximum, difference between minimum and maximum, number of local minima in the window and the number of local maxima in the same window.	3 s	4.2 s	ECG = 512 Hz Sc = 51.2 Hz	FOG Prediction	accuracy 71.3%	Cubid dataset [42]
[116]	2015	23 patients	accelerometer	1 sensor: Waist	Threshold	Freeze Index	2 s, 4 s and 6 s	-	100 Hz	FOG Detection	76% specificity and 75% sensitivity	-
[35]	2015	5 patients	IMU, ECG and SC sensors	2 sensors: chest (1), finger (1)	Frequency Features Trends	Stride duration, stride length, stance phase percentage, and LR/RL limb durations	4 s to 6 s	4 s	ECG = 512 Hz Sc = 51.2 Hz	Pre-FOG Detection	-	Cubid dataset [42]
[117]	2015	15 patients	Accelerometer Gyroscope	3 sensors: Waist (1), Trouser pocket (1), Shin (1)	AdaBoost.M1 classifier	mean, variance, standard deviation, entropy, signal energy, FI, power, root mean square, interquantile range, kurtosis, and frequency domain features power	4 s	-	200 Hz down sampled to 50 Hz	FOG Detection	Waist: (sensitivity 86%) (specificity 91.7%) Trouser pocket: (sensitivity 84%) (specificity 92.5%)	-
[118]	2014	4 patients	3D accelerometer, a 3D magnetometer and a 3D gyrometer	1 sensor: shank	Threshold	cadence and stride length	6 s	-	100 Hz	FOG Detection	sensitivity 79.5%	-
[119]	2014	20 patients	Accelerometer	1 sensor: shoe	Threshold	root-mean-squared (RMS) mean of the acceleration	0.2 s, 1.0 s, 2.0 s, 3.0 s, 3.5 s, 4.0 s, 4.5 s, 6.0 s, and 8 s	-	250 Hz	FOG Detection	sensitivity 86%, specificity 86%	-
[120]	2014	14 patients	IMU: Accelerometer +Gyroscope	7 sensors: Lower back (1) Thigh (2) Shin (2) Foot (2)	Threshold	Freeze band power	4 s	-	50 Hz	FOG Detection	-	-
[121]	2013	16 patients	Accelerometer Gyroscope	6 sensors: Wrist (2), Shin (2), Waist (1), Chest (1)	Naïve Bayes, Random Forests, and decision tree and Random Tree algorithms	Entropy, standard deviation	1 s	-	50 Hz/60 Hz	FOG Detection	sensitivity 81.94%, specificity 98.74%, accuracy 96.11% and AUC 98.6%	-
[122]	2013	25 patients	IMU:Accelerometer + Gyroscope (only accelerometer was used)	7 sensors: back (1), Thigh (2), Shank (2), Foot (2)	Threshold	Peaks at the stride and step frequencies, Freeze Index	2.5, 5, 7.5 and 10 s	-	50 Hz	FOG Identification	sensitivity 86.2%, specificity 82.4%	-
[123]	2012	10 patients	Accelerometer	3 sensors, ankle, thigh, trunk	RT, RF, NB, BN, KNN-l, KNN-2, MLP, Ada-Boost, and bagging.	Mean, entropy, standard deviation, power, and freezing Index, variance and energy.	1 s 4 s	-	64 Hz	FOG Detection	average sensitivity of 98.35% and an average specificity of 99.72%.	Daphnet [40]
[124]	2011	16 patients	Accelerometer Gyroscope	right and left wrist, right and left leg, chest and waist	DT and RF	Entropy, sampling frequency, time position, sample of the signal and probability.	1 s and 2 s	-	62.5 Hz	FOG Detection	accuracy 96.11%	-
[125]	2011	10 patients	Accelerometer Electromyographic	3 sensors: Forearm (1), Thigh (1), Skin (1)	Dynamic neural network and linear classifier	Various features designed to distinguish voluntary movements from involuntary movements.	2 s	-	1000 Hz	FOG Detection	specificity 97.3%, sensitivity 82.9%	-

DTF: directed transfer function, ICA: independent component analysis, CNN: Convolutional Neural Network, LRP: Layer-wise Relevance Propagation, LSTM: Long Short-Term Memory, KNN: k-Nearest Neighbors, LDA: Linear Discriminant Analysis, SVM: Support Vector Machine, LR: Logistic Regression, RT: Regression Tree, RF: Random Forest, NB: Naive Bayes, BN: Bayesian Network, MLP: Multilayer Perceptron, DT: decision tree, DL: Deep Learning, TCN: Temporal Convolutional Network, XGBoost: eXtreme Gradient Boosting, LightGBM: Light Gradient Boosting, NN: Neural Network, CWT: Continuous Wavelet Transform, PCA: Principal Component Analysis, RNN: Recurrent Neural Network, LRN: Layered Recurrent Network.

#### 2.4.1. Auditory Cueing Devices

In recent years, several researchers have dedicated efforts to develop devices aimed at alleviating FOG in PD patients through various forms of cueing. Bächlin et al. [50] introduced a custom-built wearable computer that provides on-demand auditory cueing during FOG episodes. The device comprises a wearable computer worn on the waist, headphones around the neck, and an acceleration sensor on the shank, all connected via Bluetooth. It features an algorithm with a lag time of less than 2 s, achieving a sensitivity of 73.1% and specificity of 81.6%. This study involved ten participants and demonstrated the potential for technology to address FOG in real time.

Similarly, Arias and Cudeiro [126] examined the effects of continuous auditory cueing using a custom-built portable device. The device, offering auditory cues via headphones, adjusts the tempo to the user’s cadence without a detailed description of the algorithm. This study expanded the participant group to include 10 PD patients with FOG, 9 PD patients without FOG, and 10 healthy subjects, exploring the broader applicability of auditory cueing across different populations.

Commercially available electronic metronomes were explored by Lee et al. [33] and McCandless et al. [38] for their effectiveness in providing rhythmic auditory cueing. These studies utilized metronomes with tempo and tone control that could be easily attached to the user, focusing on the simplicity and accessibility of cueing devices. The REMPARK system, introduced by Samá et al. [127], presents an auditory cueing system designed for the remote management of PD. Incorporating a custom-built single sensor module attached to the trunk and utilizing a smartphone for feedback, the system demonstrates FOG detection with a delay of around 3.2 s, sensitivity of 82.2%, and specificity of 92.8%. This extensive study involved 93 PD patients, demonstrating a significant effort towards scalable and effective management of FOG.

Mazilu et al. [36] developed the GaitAssist system, another auditory cueing solution facilitated by a smartphone app. With custom sensor units on each ankle, the system features a remarkably short lag time of about 0.5 s, a sensitivity of 97.1%, and a false-negative rate of 26.5%. Although tested on a smaller scale with 5 PD patients, the study emphasized the potential of integrating smartphone technology for home use. Exploring a different avenue, Zhao et al. [128] tested the effectiveness of continuous auditory cueing delivered through Google Glass. This approach utilized smart glasses worn like conventional eyewear, employing bone conduction for auditory cueing and allowing for voice and gesture control. While this study did not employ a FOG detection algorithm, it focused on the comparative effectiveness of auditory versus rhythmic visual cues among 12 PD patients.

In a more recent study, Zoetewei et al. [129] introduced the DeFOG device, which provides on-demand auditory cueing based on real-time FOG detection. Additionally, it offers feedback on physical activity, personalized to the patient’s FOG pattern, and verbal instructions for overcoming FOG episodes. The wearable technology, integrated with a smartphone for auditory cueing and feedback delivery, uses three sensors attached to the feet and chest. This trial involved 62 PD patients and highlighted the importance of achieving an optimal balance between sensitivity and specificity for real-life application, acknowledging the heterogeneity of FOG expression and the challenges of ensuring user compliance in uncontrolled settings.

#### 2.4.2. Visual Cueing Devices

In 2010, Espay et al. [130] engineered a device known as the Visual-auditory that provides dynamic visual–auditory cues through a micro-display mounted on the head and synced earphones. The device generates a virtual environment with a checkerboard pattern on the floor that moved with the user’s motion. The intervention showed a positive impact on FOG, evidenced by an improvement in the the mean score on the FOGQ decreasing from 14.2 ± 1.9 to 12.4 ± 2.5 (*p* = 0.002) across 13 PD patients.

Following this, Bryant et al. [131] introduced a walking cane equipped with a laser for continuous visual cueing. This device utilizes a laser pointer attached to a walking cane to project a static horizontal line, either red or green, and demonstrated a positive effect on reducing FOG episodes among seven PD patients. Further exploration into continuous visual cueing was conducted by Donovan et al. [132] through the U-Step walking aids, which integrates a red laser in both a walking cane and stabilizer, activated by weight or a switch on the handlebar. Their study involving 26 PD patients showed significant improvement in FOG as reflected in the FOGQ score over four weeks.

Studies continued with Buated et al. [133] presenting the LaserCane device, a walking cane equipped with a static green laser line, activated by pressing down on the ground. This device demonstrated an immediate effect on reducing both On-FOG and Off-FOG episodes among 30 participants. The wearable technology saw further advancements with Zhao et al. [128] utilizing Google Glass for optic flow and rhythmic visual cueing. Although the study involving 12 participants was inconclusive on the visual cueing’s effect on end-of-dose FOG (EoD-FOG) episodes, it opened the way for exploring smart glasses in cueing interventions.

Tang et al. [134] introduced a laser device worn on the chest that emits either a stationary or pulsating horizontal laser beam, demonstrating a significant positive effect on On-FOG episodes among 23 participants. Similarly, Ahn et al. [105] developed the Smart Gait-Aid system, leveraging Epson Moverio BT-200 smart glasses for on-demand visual cueing adjusted based on the individual’s walking pace and head orientation, achieving high sensitivity and specificity in FOG detection with minimal lag time among 10 participants.

Barthel et al. [135] took a different approach with Laser shoes, incorporating continuous visual cueing through shoes with attached lasers. Most recently, Geerse et al. [136] explored the potential of Holocue, a wearable application utilizing the HoloLens 1 mixed-reality headset for presenting on-demand, 2D and 3D holographic visual stimuli tailored to individual patient needs. Although the study involving 24 participants did not focus on a FOG detection algorithm, it emphasized the significance of device habituation and personalized cue settings. Feedback highlighted the need for further improvements in comfort and functionality, including the expansion of cue types and customizability.

#### 2.4.3. Somatosensory Stimulation Devices

In recent years, a variety of devices leveraging somatosensory stimulation have been investigated for their potential to mitigate FOG in Parkinson’s disease, each with unique characteristics and mechanisms of action.

McCandless et al. [38] tested the BodyBeat Pulsing Device, a system designed to provide rhythmic tactile feedback provided by pulsating vibrations at a customizable pace. Positioned in front of the right side of the hip, this device demonstrated a positive effect on reducing Off-FOG episodes among 20 PD patients.

In 2018, Rosenthal et al. [137] used the cueStim device, which delivers continuous electrical stimulation (ES) bursts. Worn at the waist and targeting either the hamstring or quadriceps muscles, cueStim showed a statistically significant reduction in On-FOG episodes in a group of 9 participants.

Gonçalves et al. [138] experimented with a custom-built system that offers tactile stimulation via pulsed vibration, with adjustable frequency and duration. Although encapsulated in a waistband and tested on various sites around the waist, their study centered on determining the most effective vibration frequency and duration for perception, without directly evaluating its effect on FOG amelioration among 30 participants, including both healthy individuals and those with PD.

Another solution presented by Mancini et al. [139] is the VibroGait, a somatosensory cueing device that employs vibration. Integrated with inertial sensors placed on the shins, VibroGait was found to have a positive impact on reducing Off-FOG episodes during cueing as tested on 25 PD patients.

Adding to these somatosensory interventions, Kim et al. [140] presented a novel approach with their soft robotic apparel designed to avert FOG by augmenting hip flexion. This cable-driven actuator and sensor-equipped garment significantly reduced FOG episodes during indoor walking, demonstrating its efficacy across different conditions and suggesting its potential for community use.

Klaver et al. [141] explored the efficacy of tactile cueing in addressing FOG in PD patients, through a comparison with auditory cueing. Utilizing vibrating socks to deliver tactile cues, this method claims the advantage of discretion, avoiding attention. The study enrolled 31 people with PD who underwent gait tasks in both medicated (ON) and unmedicated (OFF) states, assessing the impact of open-loop and closed-loop tactile cueing against auditory cueing and a baseline of gait without cueing. Despite the innovative approach, the results indicated that none of the cueing modalities significantly altered the percentage of time spent frozen or the number of FOG episodes on a group level. However, individual responses varied significantly, with 22 out of 31 participants experiencing improvements with cueing, underscoring the importance of personalized cueing interventions for effective FOG management in PD [141].

Each of these devices represents a step forward in the management of FOG in PD, offering various methods of somatosensory stimulation to potentially improve the quality of life for those affected by this debilitating symptom. However, it is important to note that while these devices show promise, further research is needed to fully understand their long-term efficacy, optimal settings, and application in daily life outside of controlled study environments.

## 3. Discussion

The review of the literature demonstrates the wide range of methods and algorithms used for FOG prediction. There are notable differences in the feature extraction techniques, window sizes, sampling rates, and the decision between anomaly-based (calculation) [42,95] and machine learning methods [44,77]. This diversity is a reflection of the field’s ongoing evolution and the ongoing search for the best predictive and detective models. This section provides a comprehensive discussion of various aspects of prediction and detection FOG research, including the datasets, preprocessing techniques, feature extracted, ML and non-ML algorithms, metrics utilized, and common research gaps or limitations in the literature.

### 3.1. Datasets and Preprocessing

**Figure 2:**
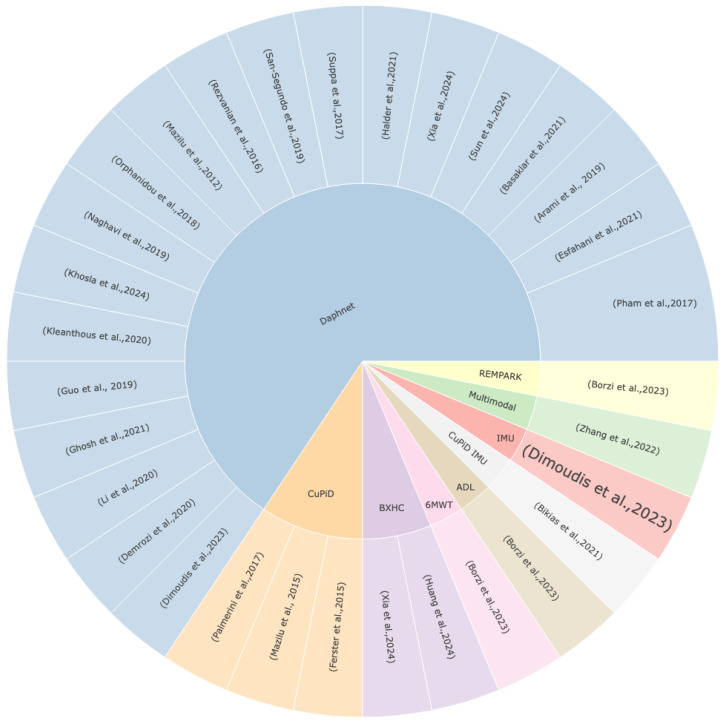
The utilization of the datasets in the literature. (Ferster et al., 2015): [35]; (Mazilu et al., 2015): [42]; (Zhang et al., 2022): [44]; (Huang et al., 2024): [54]; (Xia et al., 2024): [55]; (Khosla et al., 2024): [56]; (Sun et al., 2024): [57]; (Dimoudis et al., 2023): [60]; (Borzi et al., 2023): [65]; (Halder et al., 2021): [76]; (Esfahani et al., 2021): [80]; (Bikias et al., 2021): [81]; (Basaklar et al., 2021): [82]; (Suppa et al., 2017): [83]; (Ghosh et al., 2021): [84]; (Li et al., 2020): [85]; (Demrozi et al., 2020): [87]; (Kleanthous et al., 2020): [90]; (San-Segundo et al., 2019): [93]; (Naghavi et al., 2019): [94]; (Guo et al., 2019): [96]; (Arami et al., 2019): [98]; (Orphanidou et al., 2018): [100]; (Pham et al., 2017): [106]; (Palmerini et al., 2017): [107]; (Rezvanian et al., 2016): [114]; (Mazilu et al., 2012): [123].

The Daphnet dataset has become a mainstay in FOG prediction research as Figure 2 illustrates. Its widespread appeal can be ascribed to the extensive sensor data that it provides from patients with Parkinson’s disease, focusing on FOG. The three accelerometers in the dataset are what add value because they record subtle movements during a range of activities. It is often cited by researchers, making it appropriate for machine learning model evaluation and training. The extensive use of the Daphnet dataset in research enables insightful comparisons and promotes improvements in FOG prediction techniques [40,60,80,82,94,106,123]. Even though the Daphnet dataset is widely used, it is important to recognize its limitations. The limited size of the dataset presents difficulties that could impede the creation of models that are broadly applicable. The number of freezing events is much less than the normal ones and so requires some preprocessing.

To address the challenges posed by limitations in available datasets, researchers can adopt several strategies. Initially, the diversification of data sources should be attempted, aiming to incorporate multiple datasets to reduce the impact of individual dataset restrictions. Additionally, researchers should document the characteristics and potential biases of the datasets they utilize, enabling transparent and informed interpretation of their findings. Standardizing data collection protocols for FOG data across studies can enhance data consistency and facilitate comparisons between different datasets. Moreover, researchers can use advanced techniques for handling class imbalance, such as oversampling [97,142,143], undersampling [67,68], or employing ensemble methods [58,68,100], to ensure robust performance in classification tasks. By implementing these strategies, researchers can tackle the challenges associated with dataset limitations and class imbalance effectively, thereby improving the reliability and generalizability of their findings in FOG research.

Research indicates that EEG signals are highly effective in identifying FOG episodes [55,101,109]. Despite the challenges associated with recording, acquiring, and processing EEG data, such as expense and time consumption, its unparalleled accuracy and reliability make it indispensable in clinical settings. While the extensive nature of EEG data, often involving one or multiple electrodes, may pose logistical challenges for continuous monitoring outside clinical environments, its utility in diagnosing and managing FOG in individuals with Parkinson’s disease cannot be overstated. Therefore, while acknowledging the practical limitations for home use, EEG remains an invaluable tool for precise and timely FOG detection in clinical practice.

Regarding preprocessing techniques, the majority of papers employed similar approaches, which involved filtering out the stop or rest states and utilizing sliding windows. While some authors experimented with various window sizes [55,83,87], others relied on a single size [86,89,94], guided by recommendations from existing research. Window sizes ranged from a minimum of 0.2 s to a maximum of 10 s, with a preferred size of typically around 3 s [73]. Additionally, authors sometimes performed downsampling of the sampling rate of the dataset as necessary, depending on their specific study design [92].

The difficulty of predicting FOG is largely determined by environmental factors, data availability, and predictive models. While detecting FOG itself might not be too challenging, accurately predicting its onset well in advance can be more complicated. In terms of lead time before FOG start, the “best” outcome would vary depending on the situation and the particular prediction model or technique being applied. Table 3 illustrates how the performance of pre-FOG prediction, which is the time immediately preceding FOG episodes, differs across pre-FOG window sizes and amongst papers, as each paper used a distinct preprocessing method and feature set. But logic suggests that the patient will be informed ahead of FOG more effectively if the pre-FOG time is longer. While some studies define the pre-FOG period as the desired output [56,57,90], others use it to predict FOG episodes [67]. The duration of the pre-FOG period varies depending on the authors’ study designs and objectives.

**Table 3:** Prediction performance with different pre-FOG window sizes.

Reference	[54]		[55]		[90]	
**Performance**	**Sensitivity**	**Specificity**	**Sensitivity**	**Specificity**	**Sensitivity**	**Specificity**
1 s	69.26%	83.56%	67.02%	93.60%	-	-
2 s	70.67%	82.71%	75.77%	93.67%	91.49%	94.51%
3 s	74.79%	87.85%	81.40%	94.64%	85.11%	94.25%
4 s	72.98%	86.83%	82.88%	94.48%	76.60%	92.94%
5 s	-	-	84.61%	94.74%		

### 3.2. Features Extraction

In the literature on FOG detection and prediction, a wide array of features has been used. These features serve as crucial inputs to machine learning models or threshold algorithms. This discussion seeks to look into the significance and application of these features as indicated by their prevalence across numerous studies. Table 4 lists the features that have been used in the literature along with a description and the equation to extract each feature.

The Freeze Index (FI) is the most commonly extracted feature, utilized by a wide range of studies. Its calculation, which involves comparing the power within a freeze-associated frequency band (3–8 Hz) to the power in the locomotor band (0.5–3 Hz), serves as a direct indicator of FOG episodes. The widespread adoption of FI across studies [56,57,66,67,68,69,73,74,83,86,90,91,93,94,96,97,98,99,100,106,107,108,114,115,116,117,122,123] underlines its effectiveness in capturing the essence of freezing episodes, making it a cornerstone feature in FOG detection.

Statistical features such as standard deviation, mean, and variance capture the variability, central tendency, and dispersion of gait signal values, respectively. Their frequent use ([34,42,46,56,57,61,62,66,69,74,83,87,90,91,93,97,99,102,103,104,108,110,113,117,121,123] for Standard Deviation; [42,46,56,57,61,62,66,67,68,74,83,90,93,99,100,103,104,108,110,113,117,123] for mean; [56,61,67,68,83,86,87,93,99,103,108,117,123] for variance) highlights the importance of assessing the fundamental statistical properties of gait data when identifying patterns indicative of FOG.

Reflecting the total magnitude and distribution of energy across signal frequencies, signal energy [44,57,61,66,67,68,69,74,83,93,103,106,108,110,115,117,123] and Power [42,44,56,66,67,68,74,83,90,91,93,97,99,107,108,113,117,120,123] offers insights into the dynamic properties of gait affected by FOG. Their inclusion in numerous studies suggests a critical role in quantifying the overall intensity and frequency distribution of gait patterns disrupted by FOG. Entropy, serving as a measure of signal unpredictability or complexity, is highlighted for its utility in discerning the irregularity in gait patterns associated with FOG. Its application across studies [57,61,66,69,74,83,90,91,93,94,97,99,103,108,117,121,123,124] points to an interest in capturing the disordered nature of gait signals during freezing episodes.

Kurtosis [46,56,61,74,83,90,91,95,99,104,110,117], Skewness [46,56,61,74,83,90,91,95,104,110], and Dominant Frequency [67,68,74,86,99] provide knowledge of the distribution shape (kurtosis and skewness) and predominant frequencies (Dominant Frequency) within gait signals. Their use underscores an approach to characterize the particulars of gait dynamics affected by FOG.

**Table 4:** Features extracted by the literature.

Feature	Description/Equation	Used by
Freeze Index	The amount of power within a specific frequency band associated with freezing of gait episodes (3–8 Hz) relative to the power in the locomotor band (0.5–3 Hz) $FI=\frac{Power_{\left[3,8\right]}\left(x\right)}{Power_{\left[0.5,3\right]}\left(x\right)}$ where *x* is the signal.	[56,57,66,67,68,69,73,74,83,86,90,91,93,94,96,97,98,99,100,106,107,108,114,115,116,117,122,123]
Standard Deviation	A measure of the amount of variation or dispersion of a set of values. $\sigma =\sqrt{\frac{1}{N-1}\sum_{i=1}^{N}{\left(x_{i}-\bar{x}\right)}^{2}}$, where σ is the standard deviation, *N* is the number of observations, $x_{i}$ is the value, and $\bar{x}$ is the mean.	[34,42,46,56,57,61,62,66,69,74,83,87,90,91,93,97,99,102,103,104,108,110,113,117,121,123]
Mean	The average value of the signal. $\bar{x}=\frac{1}{N}\sum_{i=1}^{N}x_{i}$, where $\bar{x}$ is the mean, *N* is the number of observations, and $x_{i}$ is the value of the signal.	[42,46,56,57,61,62,66,67,68,74,83,90,93,99,100,103,104,108,110,113,117,123]
Power	Total power across all frequencies of interest in a signal. $TotalPower=\int_{f_{min}}^{f_{max}}PSD\left(f\right)df$, where PSD(f) is the power spectral density function, indicating the power of the signal at each frequency *f*, and $f_{min}$ and $f_{max}$ are the minimum and maximum frequencies of interest, respectively.	[42,44,56,66,67,68,74,83,90,91,93,97,99,107,108,113,117,120,123]
Entropy	A measure of the amount of uncertainty or information content in the signal. $Entropy\left(H\right)=-\sum_{i=1}^{N}p\left(x_{i}\right)\log_{2}p\left(x_{i}\right)$ where $p(x_{i})$ is the probability of occurrence of the ith value.	[57,61,66,69,74,83,90,91,93,94,97,99,103,108,117,121,123,124]
Energy	The total magnitude of the signal squared, summed over time. $Energy=\sum_{i=1}^{N}{\left(x_{i}\right)}^{2}$	[44,57,61,66,67,68,69,74,83,93,103,106,108,110,115,117,123]
Maximum	The maximum value in a given window $max\left(x\right)=max\left(x_{1},x_{2},\ldots ,x_{N}\right)$	[42,44,56,61,62,66,67,68,83,87,90,93,101]
Minimum	The minimum value in a given window $min\left(x\right)=min\left(x_{1},x_{2},\ldots ,x_{N}\right)$	[42,44,56,61,62,66,67,68,83,87,90,93,101]
Variance	A measure of the dispersion of a set of values around the mean. $\sigma^{2}=\frac{1}{N-1}\sum_{i=1}^{N}{\left(x_{i}-\bar{x}\right)}^{2}$, where $sigma^{2}$ is the variance, *N* is the number of observations, $x_{i}$ is the value, and $\bar{x}$ is the mean.	[56,61,67,68,83,86,87,93,99,103,108,117,123]
Kurtosis	It measures the ’tailedness’ of the data distribution. $kurtosis=\frac{n(n+1)}{\left(n-1)(n-2)(n-3\right)}\left(\sum_{i=1}^{n}\frac{{\left(x_{i}-\bar{x}\right)}^{4}}{SD^{4}}\right)-\frac{3{\left(n-1\right)}^{2}}{\left(n-2)(n-3\right)}$, where *n* is the number of observations, SD is the standard deviation, $x_{i}$ is the value, and $\bar{x}$ is the mean.	[46,56,61,74,83,90,91,95,99,104,110,117]
Skewness	It measures the asymmetry of the data distribution around the mean. $skewness=\frac{n}{\left(n-1)(n-2\right)}\left(\sum_{i=1}^{n}\frac{{\left(x_{i}-\bar{x}\right)}^{3}}{SD^{3}}\right)$, where *n* is the number of observations, SD is the standard deviation, $x_{i}$ is the value, and $\bar{x}$ is the mean.	[46,56,61,74,83,90,91,95,104,110]
Root Mean Square	The square root of the total sum of squares of each data in an observation. $RMSvelocity=\sqrt{\frac{1}{N}\sum_{i=1}^{N}x_{i}^{2}}$, where *N* is the number of observations, and $x_{i}$ is the value of the signal.	[34,56,66,83,90,91,99,117,119]
Range	The difference between the highest and lowest values $Range=max\left(x_{1},x_{2},\ldots ,x_{N}\right)-min\left(x_{1},x_{2},\ldots ,x_{N}\right)$, where $x_{1},x_{2},\ldots ,x_{N}$ are the values of the signal.	[34,56,61,66,74,83,91,110]
Median	The middle value in a sorted list of numbers. $median\left(x\right)=\left\{\begin{array}{cc} x_{\frac{n+1}{2}} & n=odd\\ \frac{x_{\frac{n}{2}}+x_{n_{2}+1}}{2} & n=even \end{array}\right.$	[42,44,56,83,90,93,108]
Correlation	The extent to which two variables are linearly related. $cor=\frac{\sum_{i=1}^{N}\left(x_{i}-\bar{x}\right)\left(y_{i}-\bar{y}\right)}{\sqrt{\sum_{i=1}^{N}{\left(x_{i}-\bar{x}\right)}^{2}}\sqrt{\sum_{i=1}^{N}{\left(y_{i}-\bar{y}\right)}^{2}}}$, where *N* is the number of observations, $x_{i}$ and $\bar{x}$ are the value and the mean of the first variable, and $y_{i}$ and $\bar{y}$ are the value and the mean of the second variable	[46,91,93,104,110]
Dominant Frequency	The frequency component that has the highest energy or amplitude in the frequency spectrum. $f_{dom}=argmax_{f}\left|X\left(f\right)\right|$ where |X(f)| is the magnitude of the FFT at each frequency and denotes the frequency at which |X(f)| achieves the maximum value.	[67,68,74,86,99]
Peak Height	Peak height measures the amplitude of the peak of the signal relative to zero, representing the maximum angular velocity reached in each step. PeakHeight=Height of the signal peak above zero	[34,56,74,91,98]
Stride Time	Stride time is the time interval between two successive steps on the same leg, giving an indication of the cycle of the gait. $StrideTime=t_{\mathrm{peak}\;\mathrm{subsequent}}-t_{\mathrm{peak}\;\mathrm{initial}}$	[34,88,99,102]
Zero Crossing Rate (ZCR)	Quantifies the rate at which a signal changes from positive to negative or vice versa. $ZCR=\frac{1}{n-1}\sum_{i=1}^{n-1}[\frac{1}{2}|sgn\left(x_{i+1}\right)-sgn\left(x_{i}\right)\left|\right]$, where *n* is the number of observations, $x_{i}$ is the value of the signal, and sgn() is the sign function that returns the sign of a real number.	[44,66,74,98]
Auto Regression Coefficients	The parameters in an AutoRegressive (AR) model describe the relationship between the current value of a time series and its previous values. The general form of an AR model of order p (AR(p)) can be expressed as: $X_{t}=c+\Phi_{1}X_{t-1}+\Phi_{2}X_{t-2}+,\ldots ,+\Phi_{p}X_{t-p}+\epsilon_{t}$, where $X_{t}$ is the value of the time series at time *t*, *c* is a constant, $\Phi_{1},\Phi_{2},...,\Phi_{p}$,are the auto regression coefficients, $\epsilon_{t}$ is the error term at time t, and *p* is the order of the model.	[46,93,104]
Interquartile Range (IQR)	IQR indicates the spread of the middle 50% of a dataset. $IQR=Q_{3}-Q_{1}$, where $Q_{1}$ is the first quintile (25^th^ percentile) of the dataset, and $Q_{3}$ is the third quantile (75^th^ percentile) of the dataset.	[61,93,117]
Empirical Cumulative Distribution Function (ECDF)	ECDF describes the distribution of data points in a sample. The ECDF is defined for a set of observations $X=x_{1},x_{2},\ldots ,x_{N}$. For any value, the ECDF F(t) is calculated as $F\left(t\right)=\frac{\mathrm{Number}\;\mathrm{of}\;\mathrm{element}\;\mathrm{sinX}\;\mathrm{less}\;\mathrm{than}\;\mathrm{or}\;\mathrm{equaltot}\;\mathrm{to}\;t}{N}$, where *N* is the number of observations.	[93]
Mode	The value that appears most frequently in a dataset.	[56,83,87]
Step time	Step time is the duration between one step and the subsequent step of the opposite leg, providing information about the rhythm and pace of gait. $StepTime=t_{\mathrm{peak}\;\mathrm{current}}-t_{\mathrm{peak}\;\mathrm{contralateral}}$	[34,95,102]
Cadence	The number of steps taken per minute. $cadence=\frac{\mathrm{Number}\;\mathrm{of}\;\mathrm{Steps}\;}{\mathrm{Time}\;\mathrm{in}\;\mathrm{minutes}}$	[86,115]
Length of COP	Quantifies postural stability and control. The COP trajectory length is calculated by summing the distances between successive COP positions recorded at each time step during the analysis period.	[67,68]
Mean Absolute Value (MAV)	The average of the absolute values of the observations in a dataset. $\bar{x}=\frac{1}{N}\sum_{i=1}^{N}\left|x_{i}\right|$, where *N* is the number of observations, and $x_{i}$ is the value of the signal.	[44,66]
Median Absolute Deviation (MAD)	MAD is calculated as the median of the absolute deviations from the data’s median. For a set of observations $X=x_{1},x_{2},\ldots ,x_{N}$, the MAD is given by: $MAD=median(|x_{i}-median\left(X\right)|)$	[83,93]
Peak Frequency	The frequency that has the maximum power in the Power Spectral Density (PSD) of the signal. $f_{max}=argmax_{f}\left(PSD\left(f\right)\right)$, where PSD(f) is the power spectral density function.	[83,100]
Peak Width	Peak width is determined by the width of the peak at half of its maximum power (which corresponds to the square of the amplitude), and it is proportional to the duration of the swing phase of the step. Peak Width=Durationathal f power of the peak amplitude	[34,98]
Power Spectral Entropy	Measures the disorder or complexity of a frequency spectrum Power Spectral Entropy=−P(log(P+ϵ)) where *p* is the normalized power spectral density, and ϵ is a small constant to ensure the log term is well-defined	[34,93]
Principal Harmonic Frequency	Principal Harmonic Frequency =frequency corresponding to the max (FFT amplitude)	[34,93]
Root Mean Square Velocity	The quadradic mean of the speed of the signal x in the time domain. $RMSvelocity=\sqrt{\frac{1}{N}\sum_{i=1}^{N}diffinv{\left(x_{i}\right)}^{2})}$, where *N* is the number of observations, $x_{i}$ is the value of the signal, and diffinv() is the inverse of the diff() function. The diffinv() function provides a discrete integration for a vector, a matrix or a time series object.	[74,100]

The stride time, step time, and cadence, reflecting the temporal aspects of gait, are crucial for understanding the rhythm and pace alterations induced by FOG. Their extraction from the signal shows the significance of timing irregularities as indicators of freezing episodes. Additionally, the maximum and minimum features capture the highest and lowest values within a given signal window, respectively. Their use in studies ([42,44,56,61,62,66,67,68,83,87,90,93,101]) shows an interest in identifying the peak and trough signal levels, which can specify sudden changes in gait patterns characteristic of FOG episodes. By focusing on these extremities, researchers can gain insights into the variability and stability of gait.

RMS provides a measure of the signal’s magnitude, calculated as the square root of the average squared values ([34,56,66,83,90,91,99,117,119]). This feature is important for quantifying the overall energy of the gait signal, which may fluctuate significantly during the onset and cessation of FOG episodes.

The diversity in feature extraction highlighted in the table reflects the multifaceted nature of FOG and the complexity of detecting its occurrence accurately. While some features like the Freeze Index have gained widespread acceptance for their direct correlation with FOG episodes, others offer unique perspectives on the underlying changes in gait dynamics. The disparity in the usage frequency of these features across studies may be attributed to differences in the study design, objectives, and the specific characteristics of the participant populations.

It is evident that a multifeature approach, leveraging the combined strength of various signal characteristics, may enhance the accuracy and reliability of FOG detection algorithms. Moreover, the exploration of new features, alongside innovative machine learning techniques, could unveil deeper insights into the predictive markers of FOG.

In FOG prediction and detection research, feature selection techniques like Boruta [90,100], minimum-redundancy maximum-relevance criterion (mRMR) [98], backward elimination (BE) technique [98], and Principal Component Analysis (PCA) [90] are used to extract the most important and non-redundant features from datasets. While Boruta acts as a wrapper technique and evaluates feature relevance using random forest classifiers [144], PCA preserves important information while reducing dimensionality by translating characteristics into a lower-dimensional space [145]. The aim of mRMR is to minimize redundancy among selected features while simultaneously choosing those with the highest relevance to the target variable [146]. However, until an ideal subset is found, the backward elimination method repeatedly eliminates the least important features from the feature set [147].

Natasa et al. [90] applied feature selection using Boruta to choose the best 30, 15, and 5 features, subsequently applying SVM for classification. Their results demonstrated that the accuracy was 74.63% for 30 features, 73.13% for 15 features, and 79.85% for 5 features. By ensuring that only the most informative features are used, these strategies significantly improve the performance of FOG prediction and detection algorithms and increase the efficiency of these models.

### 3.3. Evaluation Metrics

Various approaches have been used in various research papers to evaluate models for detecting and predicting FOG. Some of the research [42,124] chose accuracy as their evaluation metric in order to evaluate the overall correctness of the model. Some, on the other hand, placed more emphasis on specificity and sensitivity [46,110,122], concentrating on the model’s accuracy in identifying positive occurrences (FOG events) or negative instances (non-FOG events). Alternative measures, such as Area Under the Curve (AUC), geometric mean, and Mean Average Precision (MAP) score, were utilized by a selection of studies [59,80,89,92]. These measures provide more detailed information about the performance of the model than just accuracy, which may not be sufficient in some situations, particularly when there is a dominance of one class in the dataset. For example, sensitivity and specificity [46,110,122] offer more details about the model’s performance in various classes, which makes them especially helpful in FOG detection where erroneous positives or false negatives might have serious consequences. As a result, the use of diverse assessment metrics highlights the significance of thorough model assessment in research projects and reflects the complexity of FOG detection activities.

The performance of different models and algorithms was assessed using measures including accuracy, sensitivity, and specificity, which we looked at in our analysis of studies on FOG detection and prediction. Stem-and-leaf tables in Table 5 provide insight into the maximum accuracy, sensitivity, and specificity values attained in these articles. Remarkably, over 50% of the research showed an accuracy higher than 90%, indicating the reliability of the used techniques. Among these, one study was particularly noteworthy for achieving an accuracy of 99.50% using an ANN model [75], while another study used a threshold method, recording the lowest accuracy of 71.3% [42]. This large range of accuracy values as shown in Table 5a highlights the variety of strategies and the relative merits of different tactics. Looking more closely, we noticed that the first, second, and third quartile accuracy scores were, respectively, 68%, 90%, and 95%, with a median of 90%. This gave us some understanding of how the accuracy numbers were distributed throughout the examined publications.

The stem-and-leaf graphic in Table 5b shows how the sensitivity levels were distributed throughout the examined articles. Interestingly, more than half of the investigations showed sensitivity levels above 81%. An autoregressive moving average model was able to achieve the maximum sensitivity of 99.20% [96], demonstrating the effectiveness of this specific method. On the other hand, the study that used a CNN model had the lowest recorded sensitivity, which was 63% [72]. Additional analysis reveals that the median sensitivity was 86%, and the first, second, and third quartile sensitivities were, respectively, 81.5%, 86%, and 93%.

Table 5c shows how the specificity values were distributed throughout the examined studies. More than half of the investigations showed a specificity higher than 88%, indicating a high degree of performance for the models and algorithms used in identifying non-FOG conditions. Conventional machine learning models achieved maximum specificity of 99.72% [123], demonstrating their efficacy in this particular scenario. On the other hand, a study using a supervised machine learning model found that the lowest specificity ever recorded was 66% [113]. This wide range of specificity values illustrates how different methodological methods perform differently from one another. Furthermore, with a 90% median specificity, the first, second, and third quartile specificities were 86%, 90%, and 95%, respectively.

### 3.4. Publication Years

**Figure 3:**
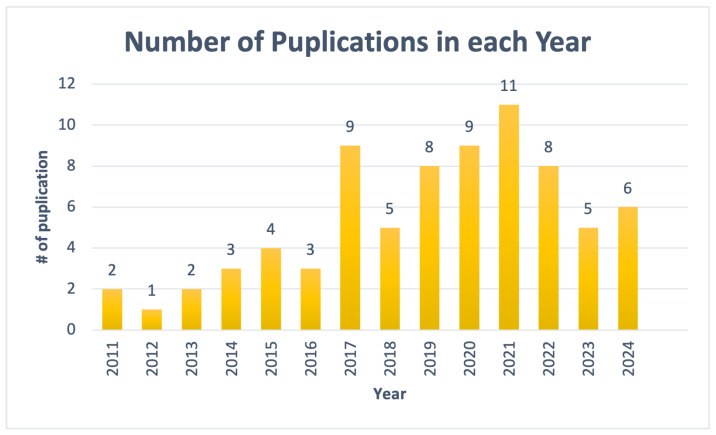
Distribution of the papers over the years.

Figure 3 shows the number of articles that were part of our meta-analysis across time, with a focus on publications from 2018 to 2022. This temporal pattern indicates a rise in interest in and research activity into FOG detection and prediction throughout this time, probably due to technological developments and an increasing understanding of the significance of treating FOG in the management of Parkinson’s disease.

### 3.5. ML vs. Non ML Approaches

In this study, we examined how well ML models and conventional non-ML methods performed in terms of prediction and detection of FOG. Considering the sophisticated capabilities linked to AI-driven techniques, this comparison is essential. Compared to conventional mathematical techniques that relied on feature extraction and threshold-based comparisons [73,83,105,106,108], machine learning models [34,56,60,75,76] offer a more automated and occasionally more efficient solution. In order to thoroughly evaluate the performance difference between these approaches, we compared the best-performing machine learning models (ML models) listed in Table 6 with non-ML techniques using a statistical z-test and *p*-test.

Notably, feature extraction and comparison against predetermined thresholds are common non-ML methods. Although successful, these methods require experience to reduce the possibility of errors. The null hypothesis is that both ML and non-ML approaches perform similarly in terms of accuracy. Our analysis’ findings showed that the z-value was 1.249 and the matching *p*-value was 0.21138, suggesting that we cannot reject the null hypothesis, and there is no statistically significant difference in accuracy between the methods. This suggests that while ML models provide increased automation and efficiency, non-ML techniques have also demonstrated notable improvements in FOG detection and prediction performance. Moreover, Table 5 offers information about the datasets that the best-performing models used, with the Daphnet dataset being the most often used. This emphasizes the Daphnet dataset’s importance and broad applicability in FOG research, as well as its critical role in promoting developments in this field.

**Table 6:** Accuracy of the top ML models against non-ML approaches using the z-test and *p*-test.

ML	Dataset	Accuracy	Non-ML Approach	Dataset	Accuracy	z-Value/*p*-Value
ANN [75]	Daphnet	99.5%	Threshold [73]	custom	99.7%	1.24975/0.21138
KNN, SVM and MLP [76]	Daphnet	98.92%	Ad hoc algorithm [83]	custom	98.51%	
CNN [60]	IMU	98.6%	Threshold [111]	custom	97.57%	
CNN [60]	Daphnet	98.1%	Adaptive Synthetic sampling algorithm [97]	custom	97.4%	
RF [56]	Daphnet	96.5%	Anomaly score detector [108]	Daphnet	94%	
DT and RF [124]	custom	96.11%	Threshold [105]	custom	92.86%	
DT and SVM [34]	custom	95.5%	Time-frequency domain analysis [106]	Daphnet	92%	

### 3.6. System Overview

**Figure 4:**
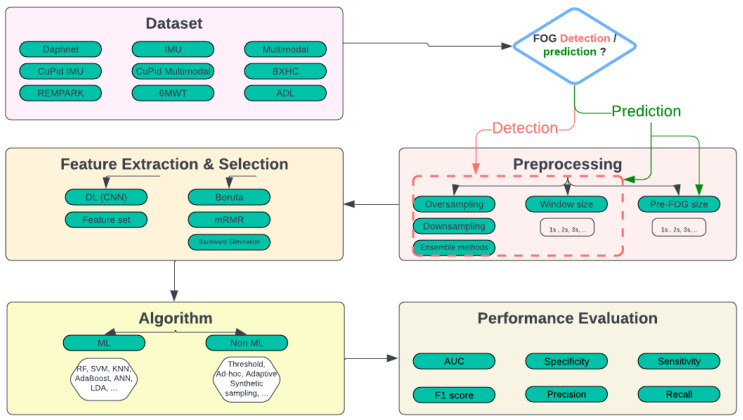
FOG detection/prediction system overview.

Following a discussion of the separate steps of the existing FOG prediction/detection systems, Figure 4 shows the overall flowchart that cohesively integrates and connects all of these stages. From data selection to classification and evaluation, this flowchart acts as a step-by-step guide for the entire process.

Choosing an acceptable dataset is the first step in constructing a FOG prediction/ detection system. Some publicly available datasets collected from PD patients are Daphnet [40], IMU [41], CuPid IMU [43], CuPid multimodal [42], multimodal [44], BXHC [45], REMPARK [46], 6MWT [47], and ADL [48]. Subsequently, researchers need to indicate if their main goal is to detect or predict FOG. This choice has a significant effect on the next steps, particularly the preprocessing methods. Preprocessing for FOG detection may involve class imbalance management using techniques like ensemble approaches [58,68,100], downsampling [67,68], and oversampling [97,142,143]. Additionally, segmenting the signal into windows and experimenting with single or multiple window sizes can optimize detection accuracy. The literature reported window sizes ranging from 0.2 s to 10 s, with a common desired size of 3 s [73]. However, for FOG prediction, an additional preprocessing step involves defining a pre-FOG period with a specific size to capture early indicators of FOG. Nevertheless, it makes sense that if the pre-FOG period is longer, the patient will be informed ahead of FOG more effectively.

Feature extraction is the next step after preprocessing. Features can be extracted manually utilizing pre-existing features from the literature or newly hypothesized features that may be useful for FOG detection or prediction [61,62], or by employing deep learning techniques like CNN [71,81,85], which automatically extract relevant features. It is critical to choose the most significant features because doing so improves performance and lowers computational time [90,98,100].

Subsequently, researchers select between non-ML and machine learning models for categorization. Popular ML models include ANN [99], KNN [87,100], SVM [100,102], RF [93,100], and others. As an alternative, ad hoc algorithm [83] and thresholding approaches [73,111] can be used for classification jobs.

The last step is to evaluate the performance of the models. While accuracy is an important measure [61,83], in this medical problem, it is crucial to gain more insights into the performance of each class. Therefore, precision [111,112], recall [112], F1 score [55], sensitivity [81,83], specificity [81,83], MAP [59], and AUC [71,92] are also used and recommended in the literature. By utilizing these metrics, researchers can ensure that their models are robust and effective in detecting or predicting FOG in a clinical context.

### 3.7. Explainability

Explainable AI approaches in the literature constitute a significant gap. Understanding the reasoning behind an algorithm’s predictions can be gained by investigating XAI methodologies, which is essential for patients and doctors to accept and comprehend the advice and alarms. The work by Filtjens et al. [77] was the only one to use XAI during the literature review. Ref. [54] used a visual representation of the feature integrator’s multihead attention maps to show how interpretable their proposed design FOG-Net is and to shed light on how the multihead self-attention module operates.

### 3.8. Limitations and Future Directions

Examining the complexities of cueing devices for FOG in Parkinson’s disease presents a range of challenges that spans auditory and visual cueing technologies. The shift from continuous to on-demand cueing to avert habituation, despite its potential, still has technical difficulties and the challenge of user adaptation [50]. The effort to dynamically tailor cueing to the individual’s gait pattern confronts the obstacle of identifying an optimally responsive tempo [126,130]. User reluctance towards awkward and visible devices underscores the critical need for device miniaturization and discreet cueing to enhance public usability and comfort [130]. The limited scale and duration of existing studies constrain the broader applicability of findings, necessitating expansive, long-term research to affirm the efficacy of auditory cueing systems [33,126]. Moreover, user feedback about device discomfort and usability determines the importance of user-centric design principles in the development of more adaptable and comfortable auditory cueing solutions [50,126]. Similarly, visual cueing devices confront the issue of bulkiness and comfort, with the visibility of such devices raising user concerns about stigma and affecting their willingness to use these aids in daily life [130,131]. The search for visual cueing systems that adapt dynamically to the user’s movements highlights the need for advancements that can provide real-time, effective support [130,134].

Across both cueing sensory systems, several key limitations arise. There is a need for integrating user experience deeply into the technology development process, ensuring that devices are tailored to meet the preferences and requirements of individuals with PD [126,130,131]. Ethical considerations and privacy concerns related to data collection and usage necessitate robust ethical standards and privacy protection measures [130]. The prevalent reliance on limited datasets like Daphnet underscores the need for broader, more diversified data collection efforts to enhance the generalizability of research outcomes. This is coupled with a call for standardized protocols that would lend reliability and comparability to study findings [38,132]. Furthermore, the economic and accessibility barriers associated with advanced cueing devices and algorithms highlight the imperative for cost-effective and widely accessible solutions [130,134]. The transition from controlled study environments to real-world applications demands rigorous, real-life validation of technologies to ensure their practical efficacy and user adaptability [135].

Studies related to somatosensory cueing devices have encountered issues such as short duration of use, raising questions about the long-term efficacy of devices [38,137]. Additionally, the small sample sizes utilized in these studies [131,139] limit the generalizability of the findings.

Another significant barrier is the variability in the sensitivity and specificity of FOG detection algorithms, impacting the system’s acceptance. Furthermore, the comfort and wearability of devices, coupled with participants’ skepticism about the devices’ effectiveness, present substantial barriers to adoption [105,130,134]. The issues of battery life, device size, and algorithm lag time also contribute to the practical challenges in daily use [127].

Addressing the limitations within the detection and prediction of FOG research in PD involves a wide array of factors beyond the specific challenges associated with cueing devices. A critical aspect is the integration of user experience and acceptance in the development and assessment of assistive technologies. The field has seen a less-than-adequate exploration of how individuals with PD interact with, perceive, and accept these technologies. A profound understanding of usability, comfort, and overall acceptance is essential for tailoring devices to the unique needs and preferences of end users, thus enhancing their effectiveness and fostering wider adoption. Addressing the user experience and acceptance of cueing devices in PD management, a mixed methods exploration by Kenny et al. [148] looks into the needs and perspectives of individuals with PD regarding wearable technologies for disease monitoring and management. Their study, conducted with people living with PD in Munster, Ireland, underscores the importance of devices being clinically useful, user friendly, and comfortable for the wearer. Participants expressed a positive outlook towards the potential benefits of wearable devices in symptom management, especially for motor dexterity. However, they also highlighted several barriers to usage, including poor hand function, average confidence in using technology, and concerns over potential costs. Interestingly, while the study found that participants predominantly viewed wearable devices as tools for providing data to healthcare professionals, there was also an acknowledgment of the importance of these devices in offering feedback directly to the users themselves. This underscores a critical need for wearable device designs to prioritize user input to enhance compliance and adoption rates among the PD population [148].

Another important area is the ethical and privacy considerations surrounding the deployment of FOG predictive and detection devices. Given that these technologies inherently collect and analyze sensitive health data, establishing comprehensive frameworks to protect user privacy and adhere to ethical standards is paramount. Future research should prioritize these considerations to ensure the responsible use of personal data and build trust among users.

Furthermore, the review highlights a dependency on limited datasets, such as Daphnet, which, while invaluable, presents challenges in terms of generalizability and applicability across diverse patient demographics. The field would greatly benefit from the creation of larger, more varied datasets that capture a wider range of FOG episodes and patient characteristics. Additionally, the adoption of standardized protocols for data collection and annotation will bolster the reliability and comparability of research outcomes.

The cost and accessibility of advanced wearable devices and the algorithms that drive them represent significant barriers to widespread utilization. Future efforts should concentrate on developing cost-effective solutions and exploring collaborative efforts to extend access to these vital technologies to a broader segment of the PD community.

Moreover, the complexity of PD and FOG necessitates a multidisciplinary approach that integrates expertise from neurology, engineering, data science, user design, and other relevant fields. Enhancing collaboration across these disciplines will promote innovation and lead to the creation of comprehensive solutions that address both the technical and human dimensions of PD management.

Lastly, there is an urgent need for extensive validation of FOG detection and prediction models in real-world settings. Many studies are conducted in controlled environments that may not fully encapsulate the complexities and unpredictability of everyday life. Undertaking real-world testing and longitudinal studies will yield insights into the technologies’ effectiveness, durability, and practicality, guiding necessary improvements and adaptations for real-life application.

It is likely that future research will concentrate on improving the detection and prediction technologies’ accuracy and dependability. To ensure that these methods efficiently predict and respond to false positive and false negative FOG episodes, researchers will work to improve machine learning algorithms and sensor systems. Furthermore, a possible future direction is to create an extensive dataset that includes both normal walks and FOG episodes. Furthermore, algorithmic fairness will be increased by using generative adversarial networks (GANs) to address bias in the data.

Because different FOG patterns exist in the cases under examination and because lower prediction outcomes in some patients may raise concerns about the patterns, more research is needed to clarify the variations in patterns of motion and how they alter as PD severity develops. It is also worthwhile to investigate the dynamic dependence between the multimodal data and create a simple, long-term FOG monitoring technique.

It is anticipated that more smartphones and other smart devices will be integrated with FOG prediction and detection devices. This will improve accessibility and facilitate data sharing with caregivers and healthcare providers, allowing for more thorough monitoring and care. Future research into non-invasive methods like brain–computer interfaces (BCIs) is an exciting prospect. By tracking brain activity and reacting with cues or stimulation, BCIs may provide a clear and accurate means of anticipating and managing forced FOG episodes.

The user’s needs should be given top priority in future work, and patients and their caregivers should be included in the development process. Their opinions and insights can be used to develop tools and technology that are useful, easy to use, and well suited to the requirements and preferences of Parkinson’s disease patients.

## 4. Conclusions

The management and control of FOG in PD patients may be entirely improved by assistive equipment and predictive and detecting technology.

This meta-analysis provides a comprehensive overview of FOG prediction and detection methodologies, underscoring the potential of wearable sensor technology and ML approaches in improving patient outcomes in Parkinson’s Disease management. By synthesizing findings from a wide range of studies published up to 2024, this paper highlights the complexity of FOG and the challenges in its management. The comparative assessment between ML and non-ML approaches, along with the exploration of XAI, offers valuable insights into the efficacy and interpretability of predictive models. The identified gaps in the current research and recommendations for future investigations serve as a roadmap for advancing FOG research and developing patient-centered solutions. Ultimately, this study contributes to the ongoing efforts aimed at enhancing mobility and autonomy for individuals living with Parkinson’s disease.

## Figures and Tables

**Table 5:** Stem-and-leaf diagrams for accuracy, sensitivity, and specificity. Example Key: 7|1 = 71%.

Stem	Leaves	Stem	Leaves	Stem	Leaves
		6	3	6	6
7	157	7	234555567889	7	5679
8	01223555566678999999	8	12222344444666667779	8	02223345667777888889999
9	000011122233455666777888899	9	111123335445556889	9	000111123333444555777777888899
(a)		(b)		(c)	

(a) Stem-and-leaf diagram for accuracy, (b) stem-and-leaf diagram for sensitivity, (c) stem-and-leaf diagram for specificity. Color code: Black—FOG, Red—Pre-FOG, Green—Walking.

## References

[1] Bloem B.R., Okun M.S., Klein C. (2021). Parkinson’s disease. Lancet.

[2] Hayes M.T. (2019). Parkinson’s disease and parkinsonism. Am. J. Med..

[3] Rea P. (2015). Essential Clinical Anatomy of the Nervous System.

[4] Sveinbjornsdottir S. (2016). The clinical symptoms of Parkinson’s disease. J. Neurochem..

[5] Kumaresan M., Khan S. (2021). Spectrum of non-motor symptoms in Parkinson’s disease. Cureus.

[6] Parkinson J. (2002). An essay on the shaking palsy. J. Neuropsychiatry Clin. Neurosci..

[7] Schrag A., Horsfall L., Walters K., Noyce A., Petersen I. (2015). Prediagnostic presentations of Parkinson’s disease in primary care: A case-control study. Lancet Neurol..

[8] Alves G., Wentzel-Larsen T., Aarsland D., Larsen J.P. (2005). Progression of motor impairment and disability in Parkinson disease. Neurology.

[9] Le Ray D., Juvin L., Ryczko D., Dubuc R. (2011). Supraspinal control of locomotion: The mesencephalic locomotor region. Prog. Brain Res..

[10] Peterson D.S., Horak F.B. (2016). Neural Control of Walking in People with Parkinsonism. Physiology.

[11] Wang D.D., Choi J.T. (2020). Brain network oscillations during gait in Parkinson’s disease. Front. Hum. Neurosci..

[12] Lin C., Ridder M., Sah P. (2023). The PPN and motor control: Preclinical studies to deep brain stimulation for Parkinson’s disease. Front. Neural Circuits.

[13] Huang C., Chu H., Zhang Y., Wang X. (2018). Deep Brain Stimulation to Alleviate Freezing of Gait and Cognitive Dysfunction in Parkinson’s Disease: Update on Current Research and Future Perspectives. Front. Neurosci..

[14] Nutt J., Bloem B., Giladi N., Hallett M., Horak F., Nieuwboer A. (2011). Freezing of gait: Moving forward on a mysterious clinical phenomenon. Lancet Neurol..

[15] Wang M., Jiang S., Yuan Y., Zhang L., Ding J., Wang J., Zhang J., Zhang K., Wang J. (2016). Alterations of functional and structural connectivity of freezing of gait in Parkinson’s disease. J. Neurol..

[16] Safiri S., Noori M., Nejadghaderi S.A., Mousavi S.E., Sullman M.J., Araj-Khodaei M., Singh K., Kolahi A.A., Gharagozli K. (2023). The burden of Parkinson’s disease in the Middle East and North Africa region, 1990–2019: Results from the global burden of disease study 2019. BMC Public Health.

[17] World Health Organization. https://www.who.int/news-room/fact-sheets/detail/parkinson-disease.

[18] Driver J.A., Logroscino G., Gaziano J.M., Kurth T. (2009). Incidence and remaining lifetime risk of Parkinson disease in advanced age. Neurology.

[19] Miller I.N., Cronin-Golomb A. (2010). Gender differences in Parkinson’s disease: Clinical characteristics and cognition. Mov. Disord..

[20] Statistics—Parkinson.org. https://www.parkinson.org/understanding-parkinsons/statistics.

[21] Van Den Eeden S.K., Tanner C.M., Bernstein A.L., Fross R.D., Leimpeter A., Bloch D.A., Nelson L.M. (2003). Incidence of Parkinson’s Disease: Variation by Age, Gender, and Race/Ethnicity. Am. J. Epidemiol..

[22] Amboni M., Stocchi F., Abbruzzese G., Morgante L., Onofrj M., Ruggieri S., Tinazzi M., Zappia M., Attar M., Colombo D. (2015). Prevalence and associated features of self-reported freezing of gait in Parkinson disease: The DEEP FOG study. Park. Relat. Disord..

[23] Perez-Lloret S., Negre-Pages L., Damier P., Delval A., Derkinderen P., Destée A., Meissner W.G., Schelosky L., Tison F., Rascol O. (2014). Prevalence, Determinants, and Effect on Quality of Life of Freezing of Gait in Parkinson Disease. JAMA Neurol..

[24] Schaafsma J., Balash Y., Gurevich T., Bartels A., Hausdorff J.M., Giladi N. (2003). Characterization of freezing of gait subtypes and the response of each to levodopa in Parkinson’s disease. Eur. J. Neurol..

[25] Giladi N., Hausdorff J.M. (2006). The role of mental function in the pathogenesis of freezing of gait in Parkinson’s disease. J. Neurol. Sci..

[26] Giladi N., Nieuwboer A. (2008). Understanding and treating freezing of gait in parkinsonism, proposed working definition, and setting the stage. Mov. Disord..

[27] Moore O., Peretz C., Giladi N. (2007). Freezing of gait affects quality of life of peoples with Parkinson’s disease beyond its relationships with mobility and gait. Mov. Disord. Off. J. Mov. Disord. Soc..

[28] Bloem B.R., Hausdorff J.M., Visser J.E., Giladi N. (2004). Falls and freezing of gait in Parkinson’s disease: A review of two interconnected, episodic phenomena. Mov. Disord. Off. J. Mov. Disord. Soc..

[29] Hausdorff J.M., Balash Y., Giladi N. (2003). Time series analysis of leg movements during freezing of gait in Parkinson’s disease: Akinesia, rhyme or reason?. Phys. A Stat. Mech. Appl..

[30] Sweeney D., Quinlan L.R., Browne P., Richardson M., Meskell P., ÓLaighin G. (2019). A technological review of wearable cueing devices addressing freezing of gait in Parkinson’s disease. Sensors.

[31] Nonnekes J., Snijders A.H., Nutt J.G., Deuschl G., Giladi N., Bloem B.R. (2015). Freezing of gait: A practical approach to management. Lancet Neurol..

[32] Okuma Y. (2014). Practical approach to freezing of gait in Parkinson’s disease. Pract. Neurol..

[33] Lee S.J., Yoo J.Y., Ryu J.S., Park H.K., Chung S.J. (2012). The effects of visual and auditory cues on freezing of gait in patients with Parkinson disease. Am. J. Phys. Med. Rehabil..

[34] Borzì L., Mazzetta I., Zampogna A., Suppa A., Olmo G., Irrera F. (2021). Prediction of Freezing of Gait in Parkinson’s Disease Using Wearables and Machine Learning. Sensors.

[35] Ferster M.L., Mazilu S., Tröster G. (2015). Gait parameters change prior to freezing in Parkinson’s disease: A data-driven study with wearable inertial units. EAI Endorsed Trans. Ambient Syst..

[36] Mazilu S., Blanke U., Dorfman M., Gazit E., Mirelman A.M., Hausdorff J., Tröster G. (2015). A wearable assistant for gait training for Parkinson’s disease with freezing of gait in out-of-the-lab environments. ACM Trans. Interact. Intell. Syst. (TIIS).

[37] Ginis P., Nackaerts E., Nieuwboer A., Heremans E. (2018). Cueing for people with Parkinson’s disease with freezing of gait: A narrative review of the state-of-the-art and novel perspectives. Ann. Phys. Rehabil. Med..

[38] McCandless P.J., Evans B.J., Janssen J., Selfe J., Churchill A., Richards J. (2016). Effect of three cueing devices for people with Parkinson’s disease with gait initiation difficulties. Gait Posture.

[39] Nieuwboer A. (2008). Cueing for freezing of gait in patients with Parkinson’s disease: A rehabilitation perspective. Mov. Disord. Off. J. Mov. Disord. Soc..

[40] Bächlin M., Plotnik M., Roggen D., Maidan I., Hausdorff J., Giladi N., Troster G. (2010). Wearable Assistant for Parkinson’s Disease Patients with the Freezing of Gait Symptom. Inf. Technol. Biomed. IEEE Trans..

[41] Ribeiro De Souza C., Miao R., Ávila De Oliveira J., Cristina De Lima-Pardini A., Fragoso De Campos D., Silva-Batista C., Teixeira L., Shokur S., Mohamed B., Coelho D.B. (2022). A Public Data Set of Videos, Inertial Measurement Unit, and Clinical Scales of Freezing of Gait in Individuals with Parkinson’s Disease During a Turning-In-Place Task. Front. Neurosci..

[42] Mazilu S., Calatroni A., Gazit E., Mirelman A., Hausdorff J.M., Tröster G. (2015). Prediction of Freezing of Gait in Parkinson’s From Physiological Wearables: An Exploratory Study. IEEE J. Biomed. Health Inform..

[43] Mazilu S., Blanke U., Roggen D., Tröster G., Gazit E., Hausdorff J.M. Engineers meet clinicians: Augmenting Parkinson’s disease patients to gather information for gait rehabilitation. Proceedings of the 4th Augmented Human International Conference.

[44] Zhang W., Yang Z., Li H., Huang D., Wang L., Wei Y., Zhang L., Ma L., Feng H., Pan J. (2022). Multimodal Data for the Detection of Freezing of Gait in Parkinson’s Disease. Sci. Data.

[45] Li H. (2021). Multimodal dataset of freezing of gait in parkinson’s disease. Mendeley Data.

[46] Rodríguez-Martín D., Samà A., Pérez-López C., Català A., Moreno Arostegui J.M., Cabestany J., Bayés À., Alcaine S., Mestre B., Prats A. (2017). Home detection of freezing of gait using support vector machines through a single waist-worn triaxial accelerometer. PLoS ONE.

[47] Borzì L., Varrecchia M., Olmo G., Artusi C.A., Fabbri M., Rizzone M.G., Romagnolo A., Zibetti M., Lopiano L. (2019). Home monitoring of motor fluctuations in Parkinson’s disease patients. J. Reliab. Intell. Environ..

[48] Borzì L., Olmo G., Artusi C.A., Fabbri M., Rizzone M.G., Romagnolo A., Zibetti M., Lopiano L. (2020). A new index to assess turning quality and postural stability in patients with Parkinson’s disease. Biomed. Signal Process. Control.

[49] Ginis P., Heremans E., Ferrari A., Bekkers E.M., Canning C.G., Nieuwboer A. (2017). External input for gait in people with Parkinson’s disease with and without freezing of gait: One size does not fit all. J. Neurol..

[50] Bächlin M., Plotnik M., Roggen D., Giladi N., Hausdorff J.M., Tröster G. (2010). A wearable system to assist walking of Parkinson s disease patients. Methods Inf. Med..

[51] Zhang W., Han Y., Shi Y., Yan S., Song W., Cui G., Xiang J. (2023). Effects of wearable visual cueing on gait pattern and stability in patients with Parkinson’s disease. Front. Neurol..

[52] Hoogendoorn E.M., Geerse D.J., van Dam A.T., Stins J.F., Roerdink M. (2024). Gait-modifying effects of augmented-reality cueing in people with Parkinson’s disease. Front. Neurol..

[53] Vitório R., Morris R., Das J., Walker R., Mancini M., Stuart S. (2022). Brain activity response to cues during gait in Parkinson’s disease: A study protocol. PLoS ONE.

[54] Huang D., Wu C., Wang Y., Zhang Z., Chen C., Li L., Zhang W., Zhang Z., Li J., Guo Y. (2024). Episode-level prediction of freezing of gait based on wearable inertial signals using a deep neural network model. Biomed. Signal Process. Control.

[55] Xia Y., Sun H., Zhang B., Xu Y., Ye Q. (2024). Prediction of freezing of gait based on self-supervised pretraining via contrastive learning. Biomed. Signal Process. Control.

[56] Khosla A., Kumar N., Khera P. (2024). Machine learning approach for predicting state transitions via shank acceleration data during freezing of gait in Parkinson’s disease. Biomed. Signal Process. Control.

[57] Sun H., Ye Q., Xia Y. (2024). Predicting freezing of gait in patients with Parkinson’s disease by combination of Manually-Selected and deep learning features. Biomed. Signal Process. Control.

[58] Yang P.K., Filtjens B., Ginis P., Goris M., Nieuwboer A., Gilat M., Slaets P., Vanrumste B. (2024). Freezing of gait assessment with inertial measurement units and deep learning: Effect of tasks, medication states, and stops. J. Neuroeng. Rehabil..

[59] Cohen S., Rokach L. (2024). BagStacking: An Integrated Ensemble Learning Approach for Freezing of Gait Detection in Parkinson’s Disease. arXiv.

[60] Dimoudis D., Tsolakis N., Magga-Nteve C., Meditskos G., Vrochidis S., Kompatsiaris I. (2023). InSEption: A robust mechanism for predicting FoG episodes in PD patients. Electronics.

[61] Ouyang S., Chen Z., Chen S., Zhao J. Prediction of Freezing of Gait in Parkinson’s Disease Using Time-Series Data from Wearable Sensors. Proceedings of the 2023 42nd Chinese Control Conference (CCC).

[62] Mo W.T., Chan J.H. Freezing of Gait Prediction Using Deep Learning. Proceedings of the 13th International Conference on Advances in Information Technology.

[63] Howard A., Salomon A., Gazit E., L-Jevster H.C., Hausdorff J., Kirsch L., Maggie, Ginis P., Holbrook R., Karim Y.F. (2023). Parkinson’s Freezing of Gait Prediction. https://kaggle.com/competitions/tlvmc-parkinsons-freezing-gait-prediction.

[64] Bajpai R., Khare S., Joshi D. (2023). A Multimodal Model-Fusion Approach for Improved Prediction of Freezing of Gait in Parkinson’s Disease. IEEE Sens. J..

[65] Borzì L., Sigcha L., Rodríguez-Martín D., Olmo G. (2023). Real-time detection of freezing of gait in Parkinson’s disease using multi-head convolutional neural networks and a single inertial sensor. Artif. Intell. Med..

[66] Ren K., Chen Z., Ling Y., Zhao J. (2022). Recognition of freezing of gait in Parkinson’s disease based on combined wearable sensors. BMC Neurol..

[67] Pardoel S., Nantel J., Kofman J., Lemaire E.D. (2022). Prediction of freezing of gait in Parkinson’s disease using unilateral and bilateral plantar-pressure data. Front. Neurol..

[68] Pardoel S., Shalin G., Nantel J., Lemaire E.D., Kofman J. (2021). Early detection of freezing of gait during walking using inertial measurement unit and plantar pressure distribution data. Sensors.

[69] Guo Y., Huang D., Zhang W., Wang L., Li Y., Olmo G., Wang Q., Meng F., Chan P. (2022). High-accuracy wearable detection of freezing of gait in Parkinson’s disease based on pseudo-multimodal features. Comput. Biol. Med..

[70] Shi B., Tay A., Au W.L., Tan D.M.L., Chia N.S.Y., Yen S.C. (2022). Detection of Freezing of Gait Using Convolutional Neural Networks and Data From Lower Limb Motion Sensors. IEEE Trans. Biomed. Eng..

[71] O’Day J., Lee M., Seagers K., Hoffman S., Jih-Schiff A., Kidziński Ł., Delp S., Bronte-Stewart H. (2022). Assessing inertial measurement unit locations for freezing of gait detection and patient preference. J. Neuroeng. Rehabil..

[72] Naghavi N., Wade E. (2022). Towards Real-Time Prediction of Freezing of Gait in Patients with Parkinson’s Disease: A Novel Deep One-Class Classifier. IEEE J. Biomed. Health Inform..

[73] Pierleoni P., Belli A., Bazgir O., Maurizi L., Paniccia M., Palma L. (2019). A Smart Inertial System for 24h Monitoring and Classification of Tremor and Freezing of Gait in Parkinson’s Disease. IEEE Sens. J..

[74] Mesin L., Porcu P., Russu D., Farina G., Borzì L., Zhang W., Guo Y., Olmo G. (2022). A multi-modal analysis of the freezing of gait phenomenon in Parkinson’s disease. Sensors.

[75] Prado A., Kwei K., Vanegas-Arroyave N., Agrawal S.K. Identification of Freezing of Gait in Parkinson’s Patients Using Instrumented Shoes and Artificial Neural Networks. Proceedings of the 2020 8th IEEE RAS/EMBS International Conference for Biomedical Robotics and Biomechatronics (BioRob).

[76] Halder A., Singh R., Suri A., Joshi D. (2021). Predicting State Transition in Freezing of Gait via Acceleration Measurements for Controlled Cueing in Parkinson’s Disease. IEEE Trans. Instrum. Meas..

[77] Filtjens B., Ginis P., Nieuwboer A., Afzal M.R., Spildooren J., Vanrumste B., Slaets P. (2021). Modelling and identification of characteristic kinematic features preceding freezing of gait with convolutional neural networks and layer-wise relevance propagation. BMC Med. Inform. Decis. Mak..

[78] Spildooren J., Vercruysse S., Desloovere K., Vandenberghe W., Kerckhofs E., Nieuwboer A. (2010). Freezing of gait in Parkinson’s disease: The impact of dual-tasking and turning. Mov. Disord..

[79] Shalin G., Pardoel S., Lemaire E.D., Nantel J., Kofman J. (2021). Prediction and detection of freezing of gait in Parkinson’s disease from plantar pressure data using long short-term memory neural-networks. J. Neuroeng. Rehabil..

[80] Esfahani A.H., Dyka Z., Ortmann S., Langendörfer P. (2021). Impact of Data Preparation in Freezing of Gait Detection Using Feature-Less Recurrent Neural Network. IEEE Access.

[81] Bikias T., Iakovakis D., Hadjidimitriou S., Charisis V., Hadjileontiadis L.J. (2021). DeepFoG: An IMU-Based Detection of Freezing of Gait Episodes in Parkinson’s Disease Patients via Deep Learning. Front. Robot. AI.

[82] Basaklar T., Tuncel Y., Ogras U.Y. Subject-Independent Freezing of Gait (FoG) Prediction in Parkinson’s Disease Patients. Proceedings of the 2021 IEEE Biomedical Circuits and Systems Conference (BioCAS).

[83] Suppa A., Kita A., Leodori G., Zampogna A., Nicolini E., Lorenzi P., Rao R., Irrera F. (2017). L-DOPA and freezing of gait in Parkinson’s disease: Objective assessment through a wearable wireless system. Front. Neurol..

[84] Ghosh N., Banerjee I. (2021). IoT-based freezing of gait detection using grey relational analysis. Internet Things.

[85] Li B., Yao Z., Wang J., Wang S., Yang X., Sun Y. (2020). Improved deep learning technique to detect freezing of gait in Parkinson’s disease based on wearable sensors. Electronics.

[86] Zhang Y., Yan W., Yao Y., Ahmed J.B., Tan Y., Gu D. (2020). Prediction of Freezing of Gait in Patients with Parkinson’s Disease by Identifying Impaired Gait Patterns. IEEE Trans. Neural Syst. Rehabil. Eng..

[87] Demrozi F., Bacchin R., Tamburin S., Cristani M., Pravadelli G. (2020). Toward a Wearable System for Predicting Freezing of Gait in People Affected by Parkinson’s Disease. IEEE J. Biomed. Health Inform..

[88] O’Day J.J., Kehnemouyi Y.M., Petrucci M.N., Anderson R.W., Herron J.A., Bronte-Stewart H.M. Demonstration of Kinematic-Based Closed-loop Deep Brain Stimulation for Mitigating Freezing of Gait in People with Parkinson’s Disease. Proceedings of the 2020 42nd Annual International Conference of the IEEE Engineering in Medicine & Biology Society (EMBC).

[89] Shi B., Yen S.C., Tay A., Tan D.M., Chia N.S.Y., Au W. Convolutional Neural Network for Freezing of Gait Detection Leveraging the Continuous Wavelet Transform on Lower Extremities Wearable Sensors Data. Proceedings of the 2020 42nd Annual International Conference of the IEEE Engineering in Medicine & Biology Society (EMBC).

[90] Kleanthous N., Hussain A.J., Khan W., Liatsis P. (2020). A new machine learning based approach to predict Freezing of Gait. Pattern Recognit. Lett..

[91] Reches T., Dagan M., Herman T., Gazit E., Gouskova N.A., Giladi N., Manor B., Hausdorff J.M. (2020). Using wearable sensors and machine learning to automatically detect freezing of gait during a FOG-provoking test. Sensors.

[92] Sigcha L., Costa N., Pavón I., Costa S., Arezes P., López J.M., De Arcas G. (2020). Deep learning approaches for detecting freezing of gait in Parkinson’s disease patients through on-body acceleration sensors. Sensors.

[93] San-Segundo R., Torres-Sánchez R., Hodgins J., De la Torre F. (2019). Increasing robustness in the detection of freezing of gait in Parkinson’s disease. Electronics.

[94] Naghavi N., Wade E. (2019). Prediction of Freezing of Gait in Parkinson’s Disease Using Statistical Inference and Lower–Limb Acceleration Data. IEEE Trans. Neural Syst. Rehabil. Eng..

[95] Mazzetta I., Zampogna A., Suppa A., Gumiero A., Pessione M., Irrera F. (2019). Wearable sensors system for an improved analysis of Freezing of gait in Parkinson’s disease using electromyography and inertial signals. Sensors.

[96] Guo Y., Wang L., Li Y., Guo L., Meng F. (2019). The Detection of Freezing of Gait in Parkinson’s Disease Using Asymmetric Basis Function TV-ARMA Time–Frequency Spectral Estimation Method. IEEE Trans. Neural Syst. Rehabil. Eng..

[97] Naghavi N., Miller A., Wade E. (2019). Towards real-time prediction of freezing of gait in patients with Parkinson’s disease: Addressing the class imbalance problem. Sensors.

[98] Arami A., Poulakakis-Daktylidis A., Tai Y.F., Burdet E. (2019). Prediction of Gait Freezing in Parkinsonian Patients: A Binary Classification Augmented with Time Series Prediction. IEEE Trans. Neural Syst. Rehabil. Eng..

[99] Mikos V., Heng C.H., Tay A., Yen S.C., Chia N.S.Y., Koh K.M.L., Tan D.M.L., Au W.L. (2019). A Wearable, Patient-Adaptive Freezing of Gait Detection System for Biofeedback Cueing in Parkinson’s Disease. IEEE Trans. Biomed. Circuits Syst..

[100] Orphanidou N.K., Hussain A., Keight R., Lishoa P., Hind J., Al-Askar H. Predicting Freezing of Gait in Parkinsons Disease Patients Using Machine Learning. Proceedings of the 2018 IEEE Congress on Evolutionary Computation (CEC).

[101] Handojoseno A., Naik G., Gilat M., Shine J., Nguyen T., Ly Q.T., Lewis S., Nguyen H. (2018). Prediction of Freezing of Gait in Patients with Parkinson’s Disease Using EEG Signals. Stud. Health Technol. Inform..

[102] Aich S., Pradhan P.M., Park J., Sethi N., Vathsa V.S.S., Kim H.C. (2018). A validation study of freezing of gait (FoG) detection and machine-learning-based FoG prediction using estimated gait characteristics with a wearable accelerometer. Sensors.

[103] Camps J., Samà A., Martín M., Rodriguez-Martin D., Pérez-López C., Arostegui J.M.M., Cabestany J., Catala A., Alcaine S., Mestre B. (2018). Deep learning for freezing of gait detection in Parkinson’s disease patients in their homes using a waist-worn inertial measurement unit. Knowl.-Based Syst..

[104] Samà A., Rodríguez-Martín D., Pérez-López C., Català A., Alcaine S., Mestre B., Prats A., Crespo M.C., Bayés À. (2018). Determining the optimal features in freezing of gait detection through a single waist accelerometer in home environments. Pattern Recognit. Lett..

[105] Ahn D., Chung H., Lee H.W., Kang K., Ko P.W., Kim N.S., Park T. (2017). Smart gait-aid glasses for Parkinson’s disease patients. IEEE Trans. Biomed. Eng..

[106] Pham T.T., Nguyen D.N., Dutkiewicz E., McEwan A.L., Leong P.H. Wearable healthcare systems: A single channel accelerometer based anomaly detector for studies of gait freezing in Parkinson’s disease. Proceedings of the 2017 IEEE International Conference on Communications (ICC).

[107] Palmerini L., Rocchi L., Mazilu S., Gazit E., Hausdorff J.M., Chiari L. (2017). Identification of Characteristic Motor Patterns Preceding Freezing of Gait in Parkinson’s Disease Using Wearable Sensors. Front. Neurol..

[108] Pham T.T., Moore S.T., Lewis S.J.G., Nguyen D.N., Dutkiewicz E., Fuglevand A.J., McEwan A.L., Leong P.H. (2017). Freezing of Gait Detection in Parkinson’s Disease: A Subject-Independent Detector Using Anomaly Scores. IEEE Trans. Biomed. Eng..

[109] Ly Q.T., Handojoseno A.A., Gilat M., Chai R., Ehgoetz Martens K.A., Georgiades M., Naik G.R., Tran Y., Lewis S.J., Nguyen H.T. Detection of turning freeze in Parkinson’s disease based on S-transform decomposition of EEG signals. Proceedings of the 2017 39th Annual International Conference of the IEEE Engineering in Medicine and Biology Society (EMBC).

[110] Rodríguez-Martín D., Pérez-López C., Samà A., Català A., Moreno Arostegui J.M., Cabestany J., Mestre B., Alcaine S., Prats A., Cruz Crespo M.d.l. (2017). A waist-worn inertial measurement unit for long-term monitoring of Parkinson’s disease patients. Sensors.

[111] Kita A., Lorenzi P., Rao R., Irrera F. (2017). Reliable and Robust Detection of Freezing of Gait Episodes with Wearable Electronic Devices. IEEE Sens. J..

[112] Zia J., Tadayon A., McDaniel T., Panchanathan S. Utilizing Neural Networks to Predict Freezing of Gait in Parkinson’s Patients. Proceedings of the 18th International ACM SIGACCESS Conference on Computers and Accessibilit.

[113] Mazilu S., Blanke U., Calatroni A., Gazit E., Hausdorff J., Tröster G. (2016). The role of wrist-mounted inertial sensors in detecting gait freeze episodes in Parkinson’s disease. Pervasive Mob. Comput..

[114] Rezvanian S., Lockhart T.E. (2016). Towards real-time detection of freezing of gait using wavelet transform on wireless accelerometer data. Sensors.

[115] Capecci M., Pepa L., Verdini F., Ceravolo M.G. (2016). A smartphone-based architecture to detect and quantify freezing of gait in Parkinson’s disease. Gait Posture.

[116] Zach H., Janssen A.M., Snijders A.H., Delval A., Ferraye M.U., Auff E., Weerdesteyn V., Bloem B.R., Nonnekes J. (2015). Identifying freezing of gait in Parkinson’s disease during freezing provoking tasks using waist-mounted accelerometry. Park. Relat. Disord..

[117] Kim H., Lee H.J., Lee W., Kwon S., Kim S.K., Jeon H.S., Park H., Shin C.W., Yi W.J., Jeon B.S. Unconstrained detection of freezing of Gait in Parkinson’s disease patients using smartphone. Proceedings of the 2015 37th Annual International Conference of the IEEE Engineering in Medicine and Biology Society (EMBC).

[118] Coste C.A., Sijobert B., Pissard-Gibollet R., Pasquier M., Espiau B., Geny C. (2014). Detection of freezing of gait in Parkinson disease: Preliminary results. Sensors.

[119] Kwon Y., Park S.H., Kim J.W., Ho Y., Jeon H.M., Bang M.J., Jung G.I., Lee S.M., Eom G.M., Koh S.B. (2014). A practical method for the detection of freezing of gait in patients with Parkinson’s disease. Clin. Interv. Aging.

[120] Yungher D.A., Morris T.R., Dilda V., Shine J.M., Naismith S.L., Lewis S.J., Moore S.T. (2014). Temporal characteristics of high-frequency lower-limb oscillation during freezing of gait in Parkinson’s disease. Park. Dis..

[121] Tripoliti E.E., Tzallas A.T., Tsipouras M.G., Rigas G., Bougia P., Leontiou M., Konitsiotis S., Chondrogiorgi M., Tsouli S., Fotiadis D.I. (2013). Automatic detection of freezing of gait events in patients with Parkinson’s disease. Comput. Methods Programs Biomed..

[122] Moore S.T., Yungher D.A., Morris T.R., Dilda V., MacDougall H.G., Shine J.M., Naismith S.L., Lewis S.J. (2013). Autonomous identification of freezing of gait in Parkinson’s disease from lower-body segmental accelerometry. J. Neuroeng. Rehabil..

[123] Mazilu S., Hardegger M., Zhu Z., Roggen D., Tröster G., Plotnik M., Hausdorff J.M. Online detection of freezing of gait with smartphones and machine learning techniques. Proceedings of the 2012 6th International Conference on Pervasive Computing Technologies for Healthcare (PervasiveHealth) and Workshops.

[124] Tsipouras M.G., Tzallas A.T., Tripoliti E., Rigas G., Bougia P., Fotiadis D.I., Tsouli S., Konitsiotis S. (2011). On assessing motor disorders in parkinson’s disease. Proceedings of the Wireless Mobile Communication and Healthcare: Second International ICST Conference, MobiHealth 2010.

[125] Cole B.T., Roy S.H., Nawab S.H. Detecting freezing-of-gait during unscripted and unconstrained activity. Proceedings of the 2011 Annual International Conference of the IEEE Engineering in Medicine and Biology Society.

[126] Arias P., Cudeiro J. (2010). Effect of rhythmic auditory stimulation on gait in Parkinsonian patients with and without freezing of gait. PLoS ONE.

[127] Samà A., Pérez-López C., Rodríguez-Martín D., Moreno-Aróstegui J.M., Rovira J., Ahlrichs C., Castro R., Cevada J., Graça R., Guimarães V. (2015). A double closed loop to enhance the quality of life of Parkinson’s Disease patients: REMPARK system. Innov. Med. Healthc..

[128] Zhao Y., Nonnekes J., Storcken E.J., Janssen S., van Wegen E.E., Bloem B.R., Dorresteijn L.D., van Vugt J.P., Heida T., van Wezel R.J. (2016). Feasibility of external rhythmic cueing with the Google Glass for improving gait in people with Parkinson’s disease. J. Neurol..

[129] Zoetewei D., Herman T., Brozgol M., Ginis P., Thumm P.C., Ceulemans E., Decaluwé E., Palmerini L., Ferrari A., Nieuwboer A. (2021). Protocol for the DeFOG trial: A randomized controlled trial on the effects of smartphone-based, on-demand cueing for freezing of gait in Parkinson’s disease. Contemp. Clin. Trials Commun..

[130] Espay A.J., Baram Y., Dwivedi A.K., Shukla R., Gartner M., Gaines L., Duker A.P., Revilla F.J. (2010). At-home training with closed-loop augmented-reality cueing device for improving gait in patients with Parkinson disease. J. Rehabil. Res. Dev..

[131] Bryant M.S., Rintala D.H., Lai E.C., Protas E.J. (2010). A pilot study: Influence of visual cue color on freezing of gait in persons with Parkinson’s disease. Disabil. Rehabil. Assist. Technol..

[132] Donovan S., Lim C., Diaz N., Browner N., Rose P., Sudarsky L., Tarsy D., Fahn S., Simon D. (2011). Laserlight cues for gait freezing in Parkinson’s disease: An open-label study. Park. Relat. Disord..

[133] Buated W., Sriyudthsak M., Sribunruangrit N., Bhidayasiri R. (2012). A low-cost intervention for improving gait in Parknson’s disease patients: A cane providing visual cues. Eur. Geriatr. Med..

[134] Tang L., Gao C., Wang D., Liu A., Chen S., Gu D. (2017). Rhythmic laser cue is beneficial for improving gait performance and reducing freezing of turning in Parkinson’s disease patients with freezing of gait. Int. J. Clin. Exp. Med..

[135] Barthel C., Nonnekes J., Van Helvert M., Haan R., Janssen A., Delval A., Weerdesteyn V., Debû B., Van Wezel R., Bloem B.R. (2018). The laser shoes: A new ambulatory device to alleviate freezing of gait in Parkinson disease. Neurology.

[136] Geerse D.J., Coolen B., van Hilten J.J., Roerdink M. (2022). Holocue: A wearable holographic cueing application for alleviating freezing of gait in Parkinson’s disease. Front. Neurol..

[137] Rosenthal L., Sweeney D., Cunnington A.L., Quinlan L.R., OLaighin G. (2018). Sensory electrical stimulation cueing may reduce freezing of gait episodes in Parkinson’s disease. J. Healthc. Eng..

[138] Gonçalves H., Moreira R., Rodrigues A., Santos C. (2018). Finding parameters around the abdomen for a vibrotactile system: Healthy and patients with Parkinson’s disease. J. Med. Syst..

[139] Mancini M., Smulders K., Harker G., Stuart S., Nutt J.G. (2018). Assessment of the ability of open-and closed-loop cueing to improve turning and freezing in people with Parkinson’s disease. Sci. Rep..

[140] Kim J., Porciuncula F., Yang H.D., Wendel N., Baker T., Chin A., Ellis T.D., Walsh C.J. (2024). Soft robotic apparel to avert freezing of gait in Parkinson’s disease. Nat. Med..

[141] Klaver E., van Vugt J., Bloem B., van Wezel R., Nonnekes J., Tjepkema-Cloostermans M. (2023). Good vibrations: Tactile cueing for freezing of gait in Parkinson’s disease. J. Neurol..

[142] Ashfaque Mostafa T., Soltaninejad S., McIsaac T.L., Cheng I. (2021). A Comparative Study of Time Frequency Representation Techniques for Freeze of Gait Detection and Prediction. Sensors.

[143] Singaravelu M., Mubibya G.S., Almhana J. Improving Freezing of Gait Detection and Prediction Using ML and Transformers. Proceedings of the ICC 2023—IEEE International Conference on Communications.

[144] Kursa M.B., Rudnicki W.R. (2010). Feature selection with the Boruta package. J. Stat. Softw..

[145] Yang F., Liu S., Dobriban E., Woodruff D.P. (2021). How to reduce dimension with PCA and random projections?. IEEE Trans. Inf. Theory.

[146] Peng H., Long F., Ding C. (2005). Feature selection based on mutual information criteria of max-dependency, max-relevance, and min-redundancy. IEEE Trans. Pattern Anal. Mach. Intell..

[147] Nelles O. (2002). Nonlinear System Identification. Meas. Sci. Technol..

[148] Kenny L., Moore K., O’Riordan C., Fox S., Barton J., Tedesco S., Sica M., Crowe C., Alamäki A., Condell J. (2022). The views and needs of people with Parkinson disease regarding wearable devices for disease monitoring: Mixed methods exploration. JMIR Form. Res..

